# Cytokinins: Their Impact on Molecular and Growth Responses to Drought Stress and Recovery in *Arabidopsis*

**DOI:** 10.3389/fpls.2018.00655

**Published:** 2018-05-22

**Authors:** Sylva Prerostova, Petre I. Dobrev, Alena Gaudinova, Vojtech Knirsch, Niklas Körber, Roland Pieruschka, Fabio Fiorani, Břetislav Brzobohatý, Martin černý, Lukas Spichal, Jan Humplik, Tomas Vanek, Ulrich Schurr, Radomira Vankova

**Affiliations:** ^1^Laboratory of Hormonal Regulations in Plants, Institute of Experimental Botany, Czech Academy of Sciences, Prague, Czechia; ^2^Department of Experimental Plant Biology, Faculty of Science, Charles University, Prague, Czechia; ^3^IBG-2: Plant Sciences, Institute for Bio-and Geosciences, Forschungszentrum Jülich, Jülich, Germany; ^4^Laboratory of Plant Molecular Biology, Institute of Biophysics AS and CR — Central European Institute of Technology, Mendel University in Brno, Brno, Czechia; ^5^Department of Chemical Biology and Genetics, Centre of the Region Hana for Biotechnological and Agricultural Research, Institute of Experimental Botany Czech Academy of Sciences and Faculty of Science of Palacky University, Olomouc, Czechia; ^6^Laboratory of Growth Regulators, Centre of the Region Hana for Biotechnological and Agricultural Research, Institute of Experimental Botany Czech Academy of Sciences, Faculty of Science of Palacky University, Olomouc, Czechia; ^7^Laboratory of Plant Biotechnologies, Institute of Experimental Botany, Czech Academy of Sciences, Prague, Czechia

**Keywords:** abscisic acid, auxin, cytokinin, cytokinin oxidase/dehydrogenase, drought stress, isopentenyl transferase, phytohormone

## Abstract

Our phenotyping and hormonal study has characterized the role of cytokinins (CK) in the drought and recovery responses of *Arabidopsis thaliana*. CK down-regulation was achieved by overexpression of the gene for CK deactivating enzyme cytokinin oxidase/dehydrogenase (CKX): constitutive (35S:CKX) or at the stress onset using a dexamethasone-inducible *pOp/LhGR* promoter (DEX:CKX). The 35S:CKX plants exhibited slow ontogenesis and higher expression levels of stress-associated genes, e.g., *AtP5CS1*, already at well-watered conditions. CK down-regulation resulted during drought in higher stress tolerance (indicated by relatively low up-regulation of the expression of drought stress marker gene *AtRD29B*) accompanied with lower leaf water loss. Nevertheless, these plants exhibited slow and delayed recovery after re-watering. CK levels were increased at the stress onset by stimulation of the expression of CK biosynthetic gene *isopentenyl transferase* (*ipt*) (DEX:IPT) or by application of exogenous CK *meta*-topolin. After water withdrawal, long-term CK elevation resulted in higher water loss in comparison with CKX transformants as well as with plants overexpressing *ipt* driven by senescence-inducible *SAG12* promoter (SAG:IPT), which gradually enhanced CKs during the stress progression. In all cases, CK up-regulation resulted in fast and more vigorous recovery. All drought-stressed plants exhibited growth suppression associated with elevation of abscisic acid and decrease of auxins and active CKs (with the exception of SAG:IPT plants). Apart from the *ipt* overexpressers, also increase of jasmonic and salicylic acid was found.

## Introduction

Drought belongs to the most frequent abiotic stresses which worldwide reduce crop yields (Daryanto et al., 2016). This stress may occur in nearly all climatic regions. Plants had to evolve different mechanisms for sensing and responding to drought (Zwack and Rashotte, 2015). Their interactions with the environment as well as their growth and development are regulated by plant hormones (Ha et al., 2012).

Abscisic acid (ABA) is the most important hormone controlling plant water loss, and hence their water status and performance under water-limited conditions (de Ollas and Dodd, 2016). ABA induces closure of stomata, the crucial water loss regulation site, as well as stimulates substantial transcriptional changes, associated with growth suppression and activation of defense pathways. During water stress responses, ABA exhibits a complex cross-talk with other plant hormones. For example, jasmonic acid (JA) activates synergistically several branches of ABA signaling pathway (especially MYC/MYB and ANAC transcription factors; de Ollas and Dodd, 2016). Ethylene exhibits antagonism with ABA in regulation of shoot and root growth in drought (Sharp and LeNoble, 2002). Participation of salicylic acid (SA) in drought responses is indicated by elevation of this hormone after water withdrawal as well as by positive effects of exogenous SA application on plant tolerance (Miura and Tada, 2014). Brassinosteroids enhance tolerance to abiotic stresses, probably via the effect on the antioxidant system (Wani et al., 2016). Auxins can influence plant adaptation to adverse environmental conditions by control of plant growth (Rowe et al., 2016). Thus, a complex cross-talk among different phytohormones is underlying drought stress responses.

Cytokinins (CKs) have decisive impact on regulation of plant growth as well as on the stabilization of photosynthetic machinery during stress progression. Both exogenous application and modulation of CK levels were reported to have positive effect on drought tolerance (Rulcova and Pospisilova, 2001; Rivero et al., 2007). In case of exogenous CK application, it is advantageous to use aromatic CK(s), e.g., N6-benzyladenine or *meta*-topolin, as the isoprenoid ones undergo rapid degradation by CK deactivating enzymes – cytokinin oxidase/dehydrogenases (CKX). However, the widely used aromatic CK N6-benzyladenine is quickly N-glucosylated in plants and the accumulated N7- and N9-glucosides may inhibit growth (Werbrouck et al., 1996). In contrast, its hydroxylated derivative, *meta*-topolin, is deactivated by glucosylation at the side chain. The resulting O-glucoside serves as a CK storage form that can be gradually hydrolyzed, releasing the active compound (Werbrouck et al., 1996; Werbrouck, 2010) and prolonging considerably the CK effect. For these reasons, the aromatic CK *meta*-topolin was selected for our experiments.

Taking into account hormone functions in drought responses, attempts to increase stress tolerance by manipulating of plant hormone content/signaling have been intensively studied. Major attention has been paid to ABA. Both up-regulation of ABA biosynthesis (Estrada-Melo et al., 2015) and overexpression of ABA signaling component (Mao et al., 2010) proved to enhance drought tolerance. In the former case, both decreased stomata aperture and stress-induced transcriptome changes were reported, in the latter one only transcriptome changes were found. Stress tolerance was promoted also by modulations of the content or signaling of other hormones, e.g., through overexpression of the ethylene response factor *JERF1* (Zhang et al., 2010) or elevation of auxin levels (Shi et al., 2014).

Extensive attention has been paid to CKs as well. Surprisingly, both CK down- and up-regulation were reported to enhance drought tolerance (Rivero et al., 2007; Werner et al., 2010; Nishiyama et al., 2011). Down-regulation of CK content has been mostly achieved by overexpression of *cytokinin oxidase/dehydrogenase* (*CKX*; Werner et al., 2010). Constitutive *CKX* expression resulted in slow growth rate and elevated content of protective compounds, which contributed to strongly increased drought tolerance in *Arabidopsis* (Werner et al., 2001; Nishiyama et al., 2011), tobacco (Macková et al., 2013), and barley (Pospíšilová et al., 2016), manifested e.g., by higher drought survival rate.

On the other hand, increase of CK content may also help plants to tolerate drought, as demonstrated by several studies. CK elevation has been mostly achieved by overexpression of CK biosynthetic gene *isopentenyl transferase (IPT*), driven by senescence- (*SAG12*) or stress-inducible (*SARK*) promoters. Elevation of CKs in *SAG12:ipt* creeping bentgrass (Merewitz et al., 2012; Xu et al., 2016) highly increased tolerance to drought or heat, enhancing the activity of the antioxidant system. Drought tolerance was promoted by *SARK:ipt* construct in tobacco (Rivero et al., 2007, 2009, 2010), peanut (Qin et al., 2011), or cotton (Kuppu et al., 2013). CK elevation during stress progression diminished the negative stress effects on photosynthesis. In contrast, constitutive overexpression of *ipt* under *Pssu* promoter was associated with high drought sensitivity as well as disproportion of the shoot and root system (strong root suppression; Synková et al., 1999). The above mentioned findings demonstrate that the timing and extent of CK elevation exhibits a decisive impact on plant performance.

When evaluating the CK effect on drought tolerance, it should be kept in mind that transformants may have considerably changed phenotype, e.g., 35S:CKX overexpresser shows enhanced root system, dwarf shoots, changed leaf morphology and slow growth rate (Werner et al., 2010). Due to smaller leaf surface, they have lower transpiration rate, which together with lower stomata conductance results in maintenance of higher relative water content (Lubovská et al., 2014). So, the open questions remain: What are the specific functions of CKs in drought stress responses and what may be an indirect effect mediated by changed morphology?

The aim of this study has been to elucidate specific role(s) of CKs during drought stress and subsequent recovery. The impact of CK down-regulation was compared in constitutive *CKX* transformant (35S:CKX) and dexamethasone-inducible one (DEX:CKX), in order to distinguish the consequence of changed morphology from the effect of CK suppression. In parallel, CK up-regulation driven by different promoters was used to characterize the effect of timing of CK increase on the stress response – CK elevation at the stress onset (DEX:IPT or application of exogenous CK *meta*-topolin) in comparison with the stress-induced one (SAG:IPT). Complex phenotyping mapping supported by phytohormonal and transcriptomic analyses allowed characterization of the behavior of individual variants in detail.

## Materials and methods

### Experimental setup and stress conditions

Transformant lines used in this study originated from *Arabidopsis thaliana* ecotype Columbia (Col-0): *AtCKX1* overexpressing line under *35S* promoter (35S:CKX; Werner et al., 2001); dexamethasone-inducible lines *CaMV35S*>GR>*HvCKX2* expressing *CKX2* from *Hordeum vulgare* (DEX:CKX; Cerný et al., 2013), and *CaMV35S*>GR>*ipt* (pOp^BK^-*ipt*; DEX:IPT; Craft et al., 2005); as well as the senescence inducible *ipt* transformant overexpressing *ipt* from *Agrobacterium tumefaciens* under control of *SAG12* promoter (SAG:IPT; Reusche et al., 2013). Col-0 plants were used as a control (wild-type; WT).

The genotypes were evaluated in pot experiments in the PhyTec Experimental Greenhouse at the Institute of Bio- and Geosciences, Plant Sciences (IBG-2), Forschungszentrum Jülich GmbH, Germany (50°54′36″N, 6°24′49″E). Seeds were sown on a mixture of clay and high moor peat (Pikiererde type CL P, Einheitserdewerk, Germany) with the PhenoSeeder robot system (Roussel et al., 2016). After 4 days of stratification at 4°C, plants were grown in a climate chamber at 8/16 h light/dark period, at 22/18°C, and 50% humidity. The plant germination was monitored daily by an automated germination detection system over a period of 5 days. Fourteen days after sowing (DAS), 640 *Arabidopsis* plants were transplanted into pots (7 × 7 × 7 cm) filled with a mixture (0.3/0.5/0.2) of peat, sand and pumice (SoMi 513 Dachstauden, Hawita, Germany) and watered to 60% soil water capacity (soil matric potential = 0 MPa). In the case of 35S:CKX seeds, sowing was done 14 days before the other variants (due to the delayed ontogenesis), in order to compare plants of similar rosette size. During the experiment, the plants at control conditions were watered every 2 days to a soil water capacity 30% (soil matric potential = −0.03 MPa), whereas the plants subjected to drought stress were watered for the last time 25th DAS in the morning and the water was withdrawn until 37th DAS. Soil water capacity reached 10% (soil matric potential = −1.5 MPa) in the case of WT at the time point of re-irrigation (38 DAS). The optimization of drought treatment was recently described by Barboza-Barquero et al. (2015). The photosynthetic active radiation was between 350 and 450 μmol m^−2^ s^−1^ at canopy level.

Two independent biological experiments were performed in subsequent years. In each experiment, 42 plants of each experimental variant were grown under drought stress conditions, and 56 at control conditions. The plants were randomly arranged in 16 trays (5 × 8 plants) with 7 trays at drought stress conditions and nine trays at control conditions.

Phenotypic measurements started 17 DAS (31 days in the case of 35S:CKX line) using the SCREEN Chamber system which consists of a climate chamber with an installed robot. The robot delivered the trays with plants to the GROWSCREEN FLUORO (Jansen et al., 2009) to scan leaf area by RBG measurement and chlorophyll fluorescence of dark adapted plants (F_v_/F_m_) at different time points during the experiment. The projected leaf area was used for calculation of the growth rate. Leaf area images were used for determination of number of leaves, compactness, stockiness, symmetry and diameter of rosettes. Measurements were performed every morning, before spraying and watering.

**Figure 1:**
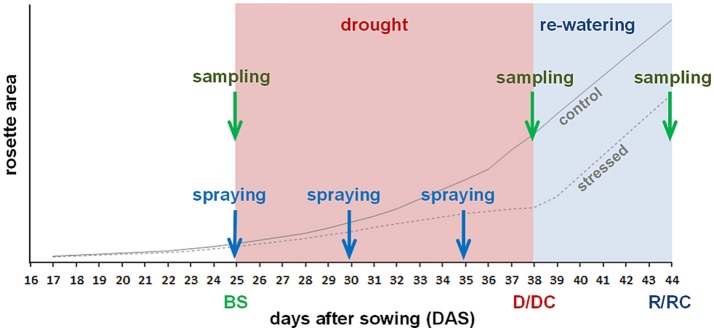
Scheme of the experiment. Drought started 25 DAS; resumption of watering was in 38 DAS. Leaf treatment by spraying was done 25, 30 and 35 DAS. Samplings were made before stress (BS), after stress (D/DC) and 6 days after re-watering (R/RC). Lines illustrate growth curves of watered control plants and drought stressed plants.

The induction of the *CKX* and *ipt* expression, respectively, in the dexamethasone-inducible lines was done by spraying with 20 μM dexamethasone (dissolved in DMSO to final concentration 0.1%, 0.01% Silwet). The spraying was carried out just before the drought stress initiation at 25 DAS, as well as on the 5th and 10th day of the stress progression. As an additional experimental variant, exogenous CK *meta*-topolin (10 μM dissolved in 0.1% DMSO with 0.01% Silwet) was sprayed on Col-0 plants at the same time as dexamethasone spraying. The other variants were sprayed with solution of 0.1% (v/v) DMSO with 0.01% Silwet. During each spraying treatment, approximately 3.2 ml was applied by sprayer on the surface of one pot and the respective plant (Figure 1).

Samples for hormone analyses, RT-qPCR, fresh and dry weight determination were taken in the morning just before the drought stress initiation (25 DAS), at the end of the drought stress period (38 DAS morning), and at the end of the recovery phase (44 DAS morning). In total 6 biological replicates were analyzed for each experimental variant. In the case of fresh and dry weight determination, each rosette was immediately weighed after cutting, dried in oven at 60°C for 24 h, and weighed again. Whole rosettes for hormone analyses and RT-qPCR were frozen in liquid nitrogen and stored at −80°C.

### Plant hormone determination

Plant hormones were purified and analyzed according to Dobrev and Kaminek (2002) and Dobrev and Vankova (2012). Samples (ca 100 mg FW) were homogenized and extracted with methanol/water/formic acid (15/4/1, v/v/v). Internal standards (10 pmol per sample) were added: ^2^H_6_-ABA, ^2^H_3_-PA, ^2^H_3_-DPA, ^2^H_4_-7OH-ABA, ^2^H_5_-ABA-GE, ^2^H_5_-*trans*Z, ^2^H_5_-*trans*ZR, ^2^H_5_-*trans*Z7G, ^2^H_5_-*trans*Z9G, ^2^H_5_-*trans*ZOG, ^2^H_5_-*trans*ZROG, ^2^H_5_-*trans*ZRMP, ^2^H_3_-DZ, ^2^H_3_-DZR, ^2^H_3_-DZ9G, ^2^H_3_-DZRMP, ^2^H_7_-DZOG, ^2^H_6_-iP, ^2^H_6_-iPR, ^2^H_6_-iPRMP, ^2^H_6_-iP7G, ^2^H_6_-iP9G, ^13^C_6_-IAA, ^2^H_2_-OxIAA, ^2^H_4_-SA, ^2^H_5_-JA (Olchemim). Extracts were purified and separated on a reverse-phase cation exchange SPE column (Oasis-MCX, Waters). The first hormone fraction was eluted with methanol (contains ABA and other acidic hormones); the second fraction, eluted with 0.35 M NH_4_OH in 70% methanol, contained CK metabolites. Both fractions were separated by HPLC (Ultimate 3000, Dionex; column Luna C18(2), 100 × 2 mm, 3 μm, Phenomenex); and the hormones were quantified using a hybrid triple quadrupole/linear ion trap mass spectrometer (3200 Q TRAP, Applied Biosystems) operated in selected reaction monitoring mode. The concentration of phytohormones was calculated relative to the corresponding internal standard or to the internal standard with a similar chemical structure.

### RT-qPCR

Total RNA was extracted with the RNeasy Plant Kit (Qiagen) from samples homogenized in liquid nitrogen with mortar and pestle (2 × 3 repetitions). RNA was treated with rDNase from NucleoSpin RNA Plant kit (Machery-Nagel). cDNA was synthesized using M-MLV Reverse Transcriptase (RNase H Minus, Point Mutant, Promega), oligo dT primers and the Protector RNase Inhibitor (Roche Applied Science). cDNA (20x diluted) was mixed with the LightCycler 480 DNA SYBR Green I Master (Roche Applied Science) and 500 nM of respective primers to a final volume 10 μl. The RT-qPCR was performed with the Light Cycler 480 (Roche Applied Science). qPCR program was set on initial denaturation (5 min, 95°C), followed by 45 cycles of primer denaturation (10 s, 95°C), annealing (10 s, 60°C) and elongation (10 s, 72°C). Relative content of RNA was calculated according to Hellemans et al. (2007). *AtUBQ10* was used as the reference gene.

Primers were designed according to sequences retrieved from TAIR database (Lamesch et al., 2012) using Primer3Plus program (Untergasser et al., 2007). The quality of primers was verified by AlleleID (PREMIER Biosoft; Apte and Singh, 2007) and the probability of folding secondary structures was predicted in mfold (Zuker et al., 1999). Primer sequences are shown in Table S1.

### Statistical analyses and calculations

Data exceeding interval of ± three standard deviations (SD) from the mean were excluded as outliers. The relative growth rate was calculated as [ln(x_2_)–ln(x_1_)]/(t_2_–t_1_), where x_1_ and x_2_ are projected leaf areas measured in t_1_ or t_2_ time points with the statistical software R (R Development Core Team, 2011). Data from hormonal analysis, RT-qPCR and growth rate determination were tested by two-sample *t*-test in the program PAST 3.01 (Hammer et al., 2001). The principal component analysis (PCA) was performed using OriginPro 2014 (http://www.originlab.com/).

## Results

### Wild-type (WT)

**Figure 2:**
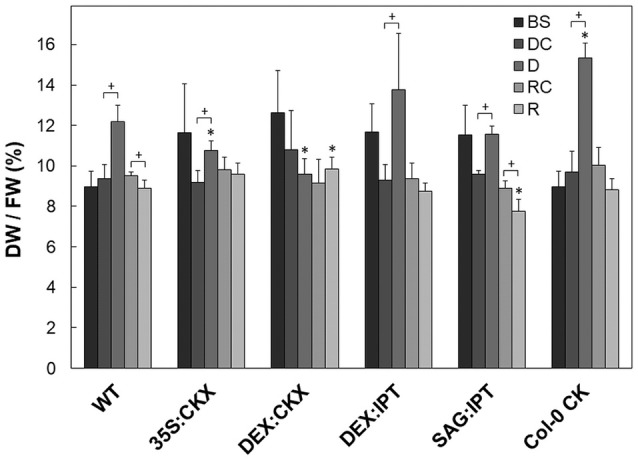
Leaf water content expressed as leaf dry weight/fresh weight ratio. The experimental variants include *Arabidopsis thaliana* wild-type (WT), 35S:CKX, DEX:CKX, DEX:IPT, SAG:IPT plants as well as Col-0 after *meta*-topolin treatment (Col-0 CK). Before stress application–BS (25 DAS), well-watered plants at the end of drought period–DC (38 DAS), drought stressed plants–D (38 DAS), well-watered plants at the end of recovery period–RC (44 DAS), re-watered plants–R (44 DAS). Statistically significant differences (*p* < 0.05, two-sample *t*-test) between the corresponding variants of WT and transformants are marked (^*^), and between D/DC or R/RC variants within each genotype (+).

**Figure 3:**
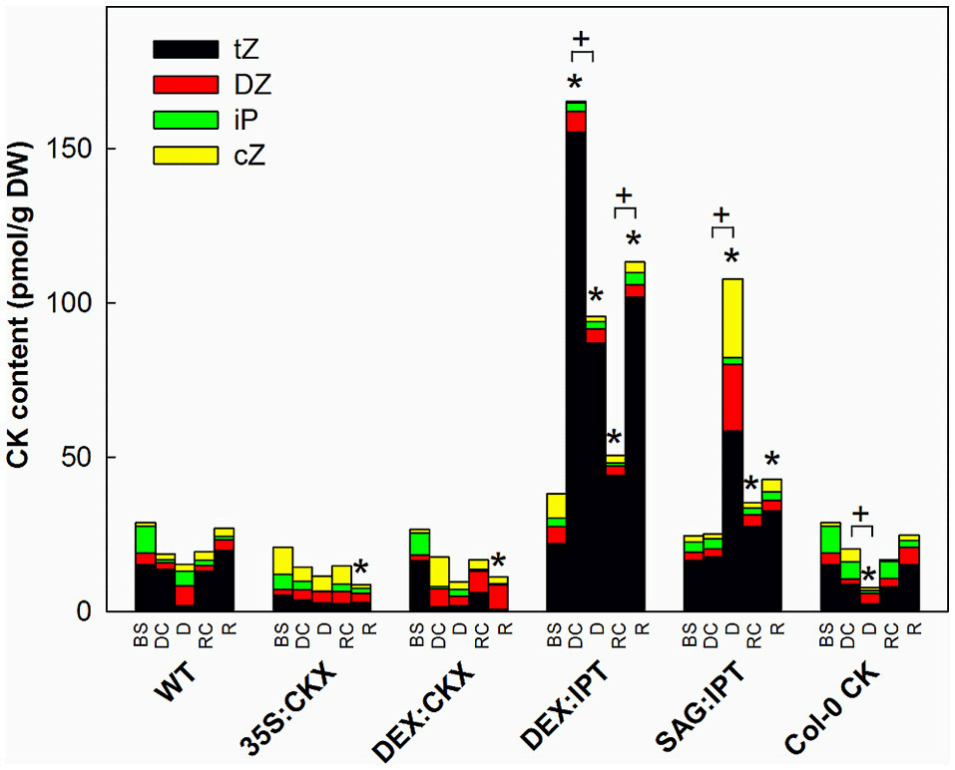
The content of active cytokinins (CKs). Specification of genotypes and treatments is as described in Figure 2. tZ - *trans*-zeatin, DZ–dihydrozeatin, iP–isopentenyladenine, cZ–*cis*-zeatin. Data represent means (*n* = 6) in pmol per g DW. Statistically significant differences (*p* < 0.05, two-sample *t*-test) in total active CK content between the corresponding variants of WT and transformants are marked (^*^), and between D/DC or R/RC variants within each genotype (+).

The drought and recovery response of the wild-type Col-0 (WT) was characterized in detail using high throughput phenotyping system. Drought stress resulted in the decrease of leaf water content (Figure 2). Stress reaction was associated with growth suppression (Figure S1, Table 1, and Table S2). The first noticeable reduction of growth was detected after 4 days of drought stress (29 DAS), then the growth rate gradually decreased. The growth suppression was accompanied by the decrease of active CKs (by 39% after 13-day drought; Figure 3), predominantly of *trans*-zeatin (tZ). Drought caused also substantial decrease of CK ribosides (by ca 67%), CK precursors (CK phosphates; by ca 65%) and CK N-glucosides (by ca 40%; Table S4). The only active CK moderately enhanced under stress conditions was *cis*-zeatin (cZ; Figure 3), accompanied by an increase of its N- and O- glucosides. In accordance with the stress-induced decrease of active CK levels, transcription of the most abundant CK catabolic enzyme *AtCKX1* was up-regulated by drought (Table 2).

**Table 1:** The impact of drought stress on growth rate of *Arabidopsis thaliana* wild-type (WT), 35S:CKX, DEX:CKX, DEX:IPT, SAG:IPT plants and Col-0 (WT) after *meta*-topolin treatment (Col-0 CK).

**DAS**	**WT**	**35S:CKX**	**DEX:CKX**	**DEX:IPT**	**SAG:IPT**	**Col-0 CK**	
22	99.7	98.0	101.1	100.4	100.7	96.5	
23	96.5	94.8	98.0	105.7	96.7	98.0	
24	94.0	97.6	93.2	100.0	101.7	91.6	
25	106.1	96.0	103.9	92.4	99.8	95.2	
28	97.6	102.3	93.0	90.2	88.5	89.9	Drought
29	82.2	72.6	89.8	65.5	80.6	55.3	
30	71.8	50.7	79.1	64.1	87.2	68.1	
31	102.3	66.3	100.3	83.4	88.5	69.8	
32	62.3	50.9	68.7	40.5	59.6	52.2	
35	56.8	17.1	57.2	32.7	51.3	49.3	
36	27.2	8.6	68.5	14.5	23.7	21.4	
37	19.2	10.9	23.1	9.7	13.5	4.6	
38	14.7	1.1	13.3	13.0	8.5	15.0	
39	115.1	9.1	57.9	116.7	86.0	147.0	Re-watering
42	192.3	141.7	127.9	175.2	190.2	155.1	
44	177.9	138.9	123.9	215.2	164.6	203.9	

*Ratio of mean growth rate of stressed plants to mean growth rate of the corresponding non-stressed plants within each time point was expressed in %. Growth rate of each plant was calculated from rosette area in mm^2^ determined by RGB measurement (n = 15–40). Individual growth rates ± SD are shown in Table S2*.

**Table 2:** The impact of drought stress on transcription of selected genes (determined by RT-qPCR) in *Arabidopsis thaliana* wild-type (WT), 35S:CKX, DEX:CKX, DEX:IPT, SAG:IPT plants and Col-0 (WT) after *meta*-topolin treatment (Col-0 CK).

		***HvCKX2***	***IPT Agro***	***CKX1***	***IPT3***	***RD29B***	***P5CS1***	***NCED3***
WT	BS			1	1	1	1	1
	DC			0.125 ± 0.074	0.129 ± 0.013	1.363 ± 0.430	0.130 ± 0.072	0.728 ± 0.081
	D			**0.916** ± **0.617**	0.111 ± 0.058	**8.390** ± **4.535**	**1.052** ± **0.171**	**5.690** ± **2.337**
	RC			0.637 ± 0.274	0.139 ± 0.054	5.029 ± 0.632	0.003 ± 0.001	7.378 ± 0.911
	R			0.647 ± 0.327	0.305 ± 0.117	**0.757** ± **0.128**	**0.357** ± **0.128**	**4.155** ± **1.182**
35S:CKX	BS			260.999 ± 95.301^*^	1.478 ± 0.034^*^	3.471 ± 0.414^*^	1.380 ± 0.240^*^	1.503 ± 0.246^*^
	DC			NA	NA	NA	NA	NA
	D			168.162 ± 65.985^*^	0.842 ± 0.097^*^	1.225 ± 0.159^*^	0.506 ± 0.107^*^	3.936 ± 0.739
	RC			121.291 ± 42.753^*^	0.524 ± 9.7E-05^*^	7.517 ± 1.879	1.001 ± 0.163^*^	3.253 ± 0.281^*^
	R			**0.334** ± **0.182**	**0.370** ± **6.8E-05**	ND	**0.499** ± **0.097**	3.372 ± 0.318
DEX:CKX	BS			1.273 ± 0.449	0.932 ± 1.7E-04	0.843 ± 0.211	0.611 ± 0.099^*^	1.576 ± 0.136^*^
	DC	1		0.192 ± 0.083	0.470 ± 0.122^*^	0.443 ± 0.057^*^	0.453 ± 0.082^*^	1.697 ± 0.664^*^
	D	1.124 ± 0.280		0.413 ± 0.164	0.529 ± 0.081^*^	**1.808** ± 0.309^*^	0.425 ± 0.070^*^	**5.668** ± 0.787^*^
	RC	0.303 ± 0.073		0.063 ± 0.022^*^	0.068 ± 1.3E-05^*^	0.047 ± 0.012^*^	0.126 ± 0.020^*^	0.463 ± 0.040^*^
	R	**0.039** ± **0.018**		ND	ND	ND	0.095 ± 0.125	ND
DEX:IPT	BS			1.410 ± 0.497	0.746 ± 1.4E-04^*^	1.309 ± 0.327	0.911 ± 0.148	1.433 ± 0.124^*^
	DC		1	0.494 ± 0.193	0.243 ± 0.044^*^	0.768 ± 0.337	0.358 ± 0.062^*^	4.535 ± 0.449^*^
	D		**2.573** ± **1.057**	1.175 ± 0.486	**0.076** ± **0.005**	**59.850** ± 6.585^*^	**1.279** ± **0.219**	**7.027** ± **1.629**
	RC		0.599 ± 0.231	0.385 ± 0.192^*^	0.367 ± 0.117^*^	0.093 ± 0.033^*^	0.276 ± 0.051^*^	0.559 ± 0.048^*^
	R		**2.135** ± **0.757**	0.662 ± 0.310	**0.075** ± 0.009^*^	0.086 ± 0.009^*^	**0.003** ± 7.3E-04^*^	**2.804** ± **0.483**
SAG:IPT	BS		ND	0.504 ± 0.177^*^	0.756 ± 1.4E-04^*^	0.316 ± 0.079^*^	0.790 ± 0.129^*^	1.427 ± 0.123^*^
	DC		0.206 ± 0.073	0.753 ± 0.323^*^	0.310 ± 0.065^*^	1.148 ± 0.737	0.416 ± 0.097^*^	4.721 ± 1.895^*^
	D		0.283 ± 0.101	1.223 ± 0.493	0.167 ± 0.063	**5.583** ± **0.621**	0.679 ± 0.147^*^	3.676 ± 0.579
	RC		0.072 ± 0.026	0.788 ± 0.278	0.671 ± 1.2E-04^*^	0.364 ± 0.091^*^	0.373 ± 0.061^*^	1.902 ± 0.164^*^
	R		**0.607** ± **0.215**	1.520 ± 0.536^*^	**0.708** ± 1.3E-04^*^	0.566 ± 0.142	**0.657** ± 0.107^*^	**2.980** ± **0.257**
Col-0 CK	BS			1	1	1	1	1
	DC			0.712 ± 0.315^*^	0.141 ± 0.024	4.532 ± 0.539^*^	0.424 ± 0.079^*^	3.701 ± 0.497^*^
	D			0.458 ± 0.161	**0.048** ± 9.0E-06^*^	**3.671** ± 0.089^*^	0.477 ± 0.078^*^	**1.097** ± 0.272^*^
	RC			0.732 ± 0.258	0.436 ± 8.1E-05^*^	0.253 ± 0.063^*^	0.326 ± 0.053^*^	0.871 ± 0.075^*^
	R			1.203 ± 0.424	**0.755** ± 1.4E-04^*^	**0.730** ± **0.183**	**0.468** ± **0.076**	**1.327** ± 0.114^*^

HvCKX2, cytokinin oxidase/dehydrogenase 2 from Hordeum vulgare; IPT Agro, isopentenyl transferase from Agrobacterium; CKX1, cytokinin oxidase/dehydrogenase 1; IPT3, isopentenyl transferase 3; RD29B, responsive to desiccation 29B; P5CS1, δ-1-pyrroline-5-carboxylate synthase 1; NCED3, 9-cis-epoxycarotenoid dioxygenase 3. The expression was determined before stress application–BS (25 DAS); in well-watered plants at the end of drought period–DC (38 DAS); in drought stressed plants–D (38 DAS); in well-watered plants at the end of recovery period–RC (44 DAS); and in re-watered plants–R (44 DAS). Transcription values represent means ± SD (n = 6) normalized to WT before stress (25 DAS). Statistically significant (p < 0.05, two-sample t-test) differences between the corresponding variants of WT and transformants are marked (

* *), and between D/DC or R/RC variants within each genotype (bold). NA, not analyzed; ND, not detected*.

**Figure 4:**
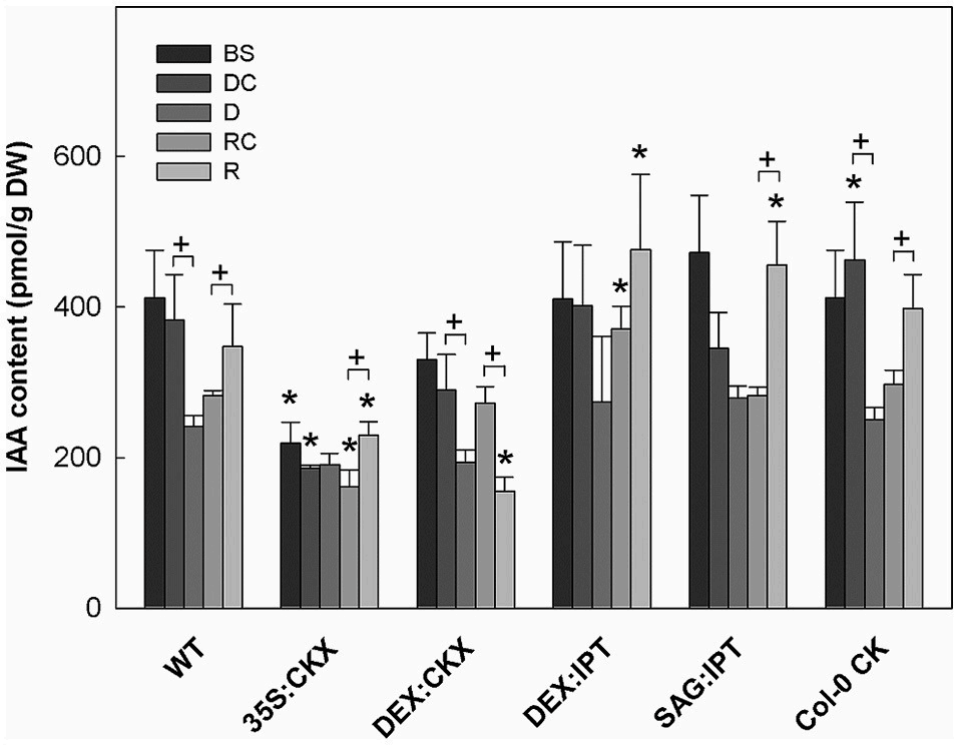
The content of auxin indole-3-acetic acid (IAA). Specification of genotypes and treatments is as described in Figure 2. Data represent means ±SD (*n* = 6) in pmol per g DW. Statistically significant differences (*p* < 0.05, two-sample *t*-test) in IAA content between the corresponding variants of WT and transformants are marked (^*^), and between D/DC or R/RC variants within each genotype (+).

**Figure 5:**
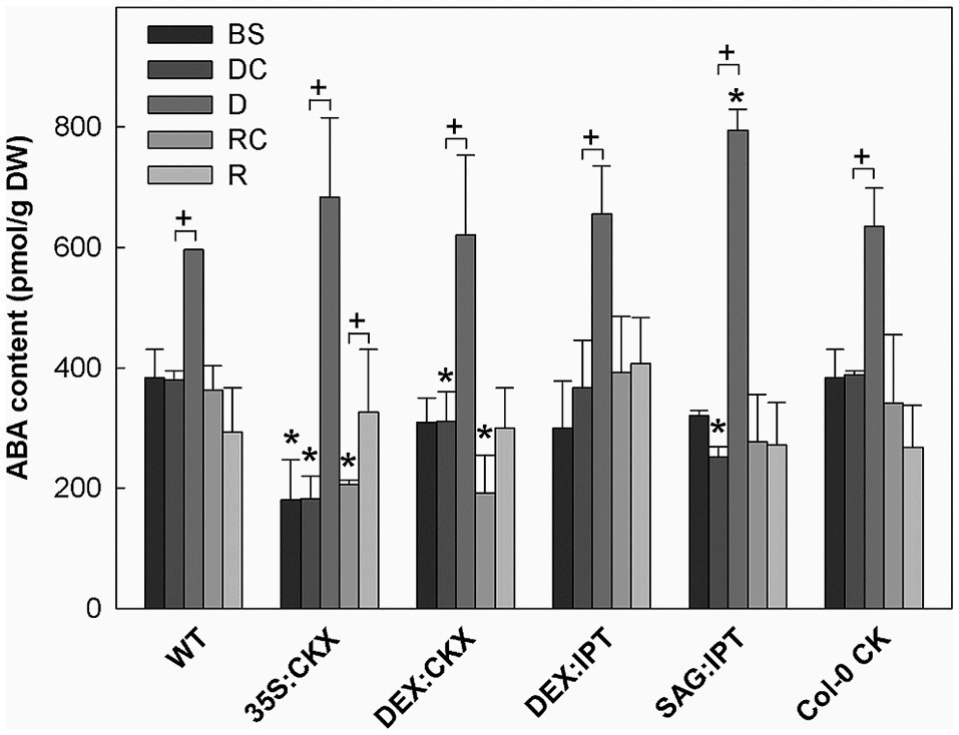
The content of abscisic acid (ABA). Specification of genotypes and treatments is as described in Figure 2. Data represent means ±SD (*n* = 6) in pmol per g DW. Statistically significant differences (*p* < 0.05, two-sample *t*-test) in ABA content between the corresponding variants of WT and transformants are marked (^*^), and between D/DC or R/RC variants within each genotype (+).

**Figure 6:**
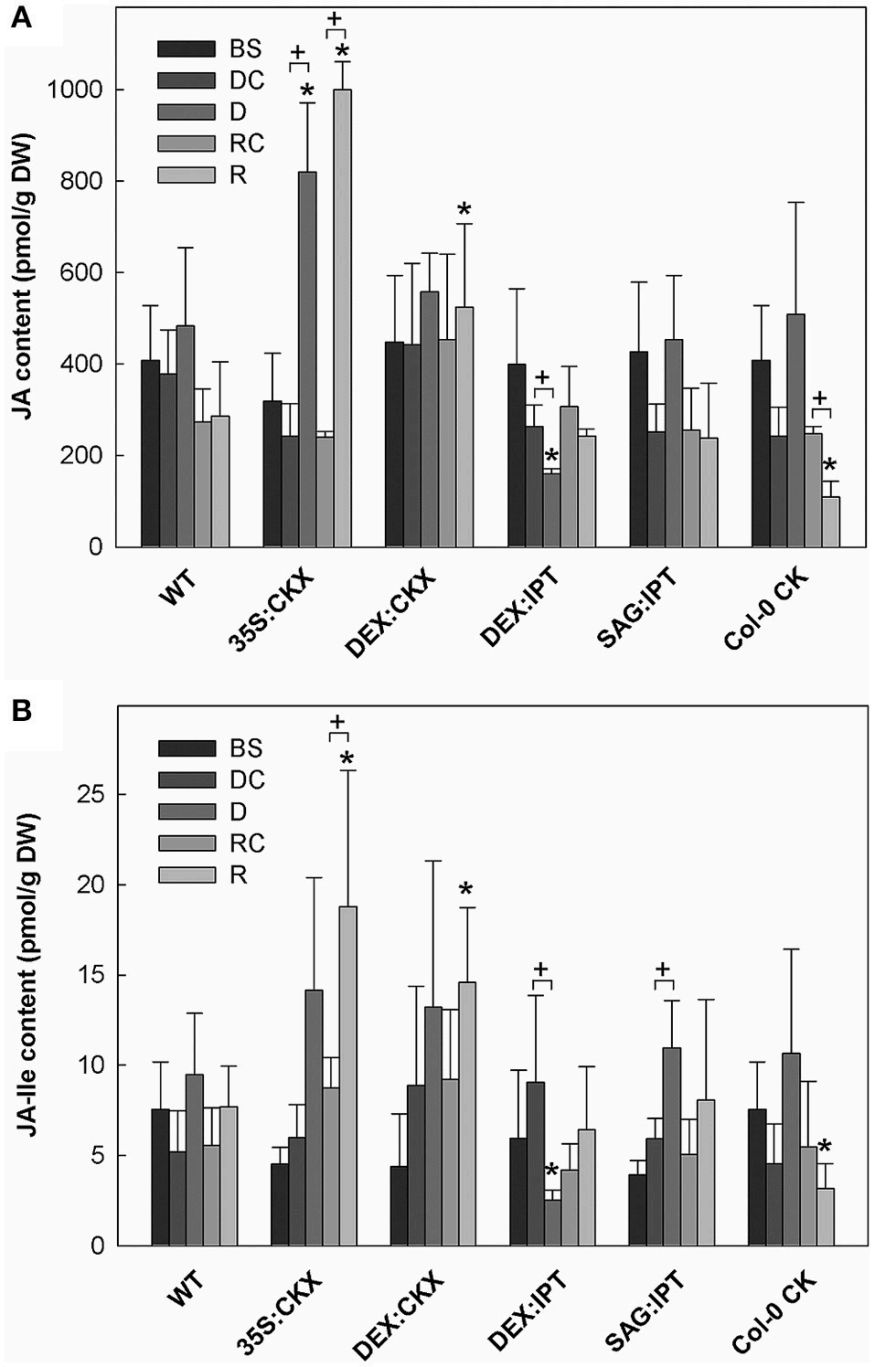
The content of **(A)** jasmonic acid (JA); and **(B)** jasmonate-isoleucine (JA-Ile). Specification of genotypes and treatments is as described in Figure 2. Data represent means ±SD (*n* = 6) in pmol per g DW. Statistically significant differences (*p* < 0.05, two-sample *t*-test) in JA and JA-Ile content between the corresponding variants of WT and transformants are marked (^*^), and between D/DC or R/RC variants within each genotype (+).

**Figure 7:**
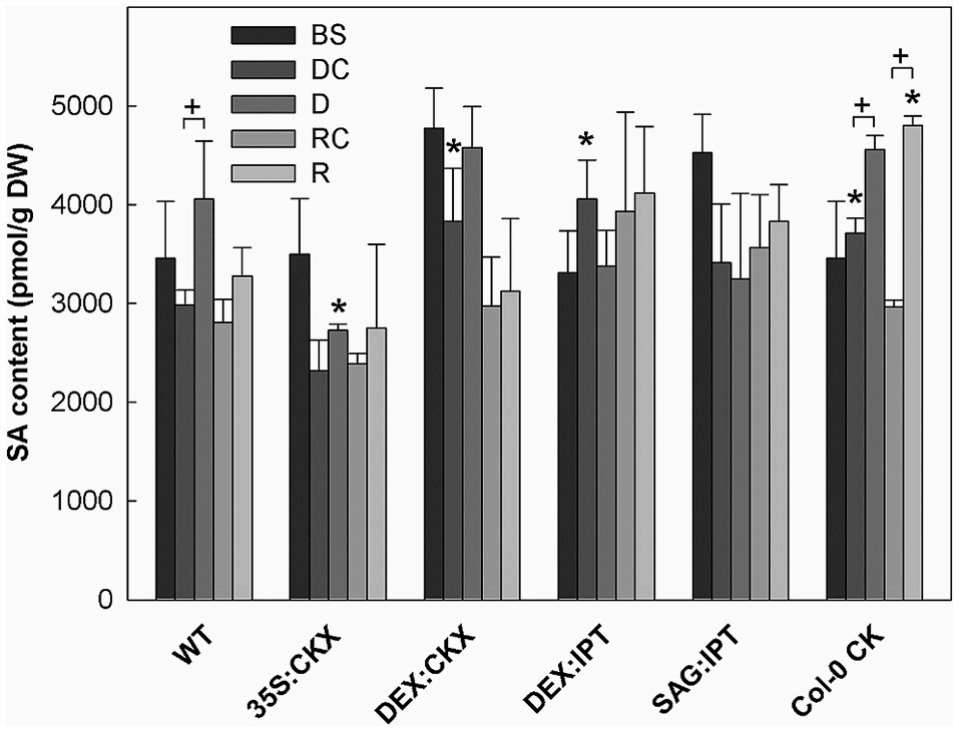
The content of salicylic acid (SA). Specification of genotypes and treatments is as described in Figure 2. Data represent means ±SD (*n* = 6) in pmol per g DW. Statistically significant differences (*p* < 0.05, two-sample *t*-test) in SA content between the corresponding variants of WT and transformants are marked (^*^), and between D/DC or R/RC variants within each genotype (+).

**Figure 8:**
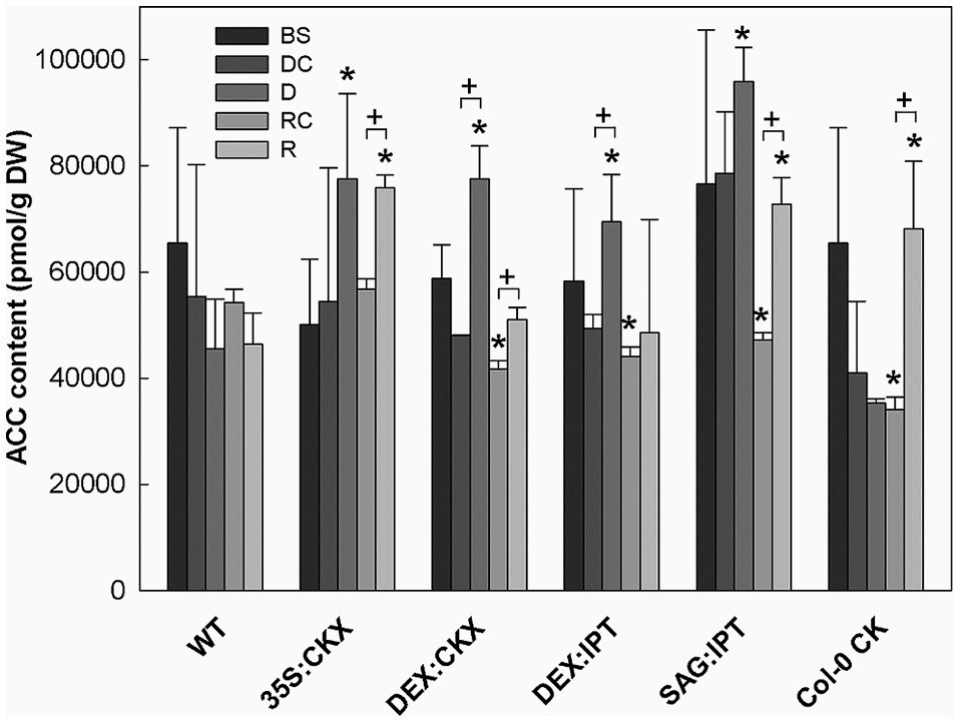
The content of the ethylene precursor aminocyclopropane carboxylic acid (ACC). Specification of genotypes and treatments is as described in Figure 2. Data represent means ±SD (*n* = 6) in pmol per g DW. Statistically significant differences (*p* < 0.05, two-sample *t*-test) in ACC content between the corresponding variants of WT and transformants are marked (^*^), and between D/DC or R/RC variants within each genotype (+).

Growth suppression was also associated with down-regulation of auxin indole-3-acetic acid (IAA; Figure 4) and simultaneous elevation of IAA catabolites 2-oxindole-3-acetic acid (OxIAA; Table S5) and its glucosyl ester (not shown), which due to biological variation did not reach statistical significance (*p* < 0.05). Similarly to IAA, the levels of another, weaker auxin phenylacetic acid also decreased (to minor extent) following the water withdrawal (Table S5). Stress response involved strong up-regulation of ABA (Figure 5), as well as of ABA catabolites: phaseic acid, dihydrophaseic acid, and 9′-hydroxy-ABA (Table S5). ABA levels correlated well with up-regulated transcription of the gene for the rate-limiting biosynthetic enzyme *9-cis-epoxycarotenoid dioxygenase NCED3* (Table 2). Drought resulted in mild elevation of JA as well as of its active conjugate jasmonate-isoleucine (JA-Ile; Figure 6). Significant increase was observed in the case of salicylic acid (SA; Figure 7), while no significant change was observed in the level of the ethylene precursor aminocyclopropane carboxylic acid (ACC; Figure 8). Expression of two selected drought stress marker genes, *responsive to desiccation 29B* (*AtRD29B*) and δ*-1-pyrroline-5-carboxylate* synthase (*P5CS1*) were strongly up-regulated in drought (Table 2).

Re-watering was accompanied by vigorous growth, at a rate exceeding the growth rate of the corresponding control plants (reaching about 180% of control; Table 1). Growth rate acceleration was accompanied by high levels of tZ and *trans*-zeatin riboside (tZR), as well as of IAA, generally high above those in the corresponding controls (Figures 3, 4). Increase of tZ-type CKs corresponded with up-regulation of *AtIPT3* due to re-watering (Table 2). ABA content was substantially diminished (Figure 5). However, the levels of the other analyzed hormones JA, JA-Ile, SA and ACC returned to control values (Figures 6–8).

### Constitutive CK down-regulation (35S:CKX)

At well-watered conditions, *AtCKX1* overexpression under *35S* promoter led to ca 150-fold increase of its transcript level in comparison with WT (Table 2). This resulted in substantially lowered levels of active CKs, especially of tZ and isopentenyladenine (iP; Figure 3), as well as of their transport forms – tZR and isopentenyladenosine (iPR; Table S4). Surprisingly, the content of cZ was higher than in WT. The enhanced *AtCKX1* expression resulted in strong down-regulation of the levels of all CK precursors (CK phosphates; to 14 % of WT) as well as of CK deactivation products — CK N- and O-glucosides (to ca 6 and 31% of WT, respectively; Table S4). Total CK levels reached only 20% of WT. Expression of *AtIPT3* was the highest from the followed genotypes (Table 2). Down-regulation of CKs was also associated with diminished content of other hormones — IAA (to ca 53% of WT), ABA (to ca 47%), to minor extent also of JA and JA-Ile (to ca 78%; Figures 4–6). Strong down-regulation of active CKs had severe impact on plant morphology – decrease of the growth rate (to ca 50% of WT; Tables S2, S3), smaller size of the rosettes, significantly higher compactness (wider blades and shorter petioles) and increase of leaf thickness. The basal expression of stress-associated genes, *AtRD29B* and *P5CS1*, was up-regulated in comparison with WT (Table 2).

Drought response of 35S:CKX plants involved slight further decrease of the growth rate (Table 1), low transcription of dehydration-responsive gene *AtRD29B* and proline-biosynthetic gene *AtP5CS1* (Table 2) as well as relatively lower water loss (by 20% in comparison with watered control at the end of drought, *vs*. 23% in the case of WT; Figure 2). Stress had relatively minor effect on rosette area at the early phase of the drought response and growth rate was maintained (Table 1, Table S2). Fifth day after stress initiation, the growth rate of 35S:CKX plants started decreasing faster compared with the other genotypes. Higher drought stress tolerance was associated with enhanced quantum efficiency of photosystem II (F_v_/F_m_) in comparison with stressed WT (Figure S2). Drought had relatively low impact on active CKs (decrease by ca 20% of well-watered 35S:CKX plants; Figure 3), as well as CK ribosides (by ca 30%; Table S4). Stress-induced down-regulation of CK precursors was only negligible. Transcription of *AtIPT3* was relatively high in drought (Table 2). No significant change was observed in IAA content in comparison with well-watered conditions, which was still lower than in the other drought stressed variants (Figure 4). ABA was induced by drought to the level comparable to those reached in the other genotypes, in spite of considerably lower value under control conditions (Figure 5). Drought resulted in substantial elevation of JA and to minor extent also of its active conjugate JA-Ile (Figure 6). Only slight elevation was found in the case of SA (Figure 7). Elevation of ACC was the most profound from all genotypes (Figure 8).

Growth re-initiation during recovery was in 35S:CKX plants much slower and smaller in comparison with the other genotypes (substantial delay by 2 days; Table 1, Table S2). After 7 days, the growth rate reached about 37% of recovered WT (Table S3B). After re-watering, the levels of active CKs were even lower than in drought due to the strong decrease of cZ (Figure 3). Only CK precursors (CK phosphates) increased after re-watering by about ca 90% in comparison with drought-stressed plants (Table S4). The content of IAA was elevated moderately above the level in the corresponding control (Figure 4). ABA, ACC and especially JA and JA-Ile content stayed elevated well above the control level during the whole followed period (Figures 5, 6, 8).

### CK down-regulation induced at the stress onset (DEX:CKX)

Morphology of DEX:CKX plants was indistinguishable from WT before the stimulation of *HvCKX2* expression at the stress onset. Up-regulation of *HvCKX2* expression by dexamethasone in both well-watered and drought-stressed DEX:CKX plants led to substantial decrease of the growth rate (in the case of watered plants to ca 80% of watered WT; in the case of stressed plants to ca 72% of stressed WT; Table S3). Growth suppression at the end of stress was 87% in comparison with the activated control plants (Table 1). Up-regulation of *HvCKX2* expression was associated with strong reduction of tZ (in well-watered conditions and in drought to 9 and 15% of non-induced plants before stress, respectively; Figure 3), tZR (to ca 51 and 39%, respectively) and of all CK phosphates (to ca 17 and 13%, respectively; Table S4). Also CK N- and O-glucosides were substantially decreased (Table S4). Stimulated *HvCKX2* expression resulted in strong suppression of endogenous *AtCKX1* transcription (which was further promoted by drought), as well as in relatively high transcription of *AtIPT3*, not affected by stress (Table 2). CK suppression by *HvCKX2* overexpression was accompanied by mild decrease of IAA, which was substantially promoted by drought (Figure 4). The impact of drought on elevation of ABA or ACC content was high (Figures 5, 8). Mild positive trend was found in the regulation of SA and JA/JA-Ile levels, not reaching statistical significance (Figures 6, 7). Nevertheless, transcription of the stress marker genes *AtRD29B* and *AtP5CS1* was low, similar to 35S:CKX transformant (Table 2). The water loss was the lowest in comparison with the other tested genotypes (by only ca 15% in comparison with well-watered control; Figure 2).

Re-watering resulted in only slight up-regulation of active CKs in comparison with the corresponding drought-stressed plants (by ca 17% at the end of recovery period; Figure 3), while CK ribosides were elevated much more (by ca 129%; Table S4). The growth reactivation was thus much slower and smaller than in WT. The growth rate reached just about 123% of the corresponding control at the end of recovery period (Table 1). No up-regulation of IAA in comparison with stress conditions was observed (Figure 4). ABA, JA-Ile and ACC content remained slightly higher than in the corresponding control (Figures 5, 6B, 8). JA, similarly to SA, was close to the control level (Figures 6A, 7).

### CK up-regulation induced at the stress onset (DEX:IPT)

After induction with dexamethasone, the DEX:IPT transformants expressed CK biosynthetic gene *isopentenyl transferase* (*ipt*) from *Agrobacterium tumefaciens*. Transcription of endogenous *AtIPT3* was down-regulated (Table 2). The activation of *ipt* gene led in control plants to huge increase of tZ (ca 10-times compared to WT; Figure 3), tZR (ca 13-times) and tZR phosphate (ca 28-times), as well as of dihydrozeatin (DZ) riboside phosphate (ca 50-times), CK N- (ca 7-times) and O-glucosides (ca 14-times; Table S4). Stimulation of *ipt* under well-watered conditions resulted in the mild increase of ABA (as well as of its metabolites); while the other stress hormones JA and ACC were slightly decreased (Figures 5, 6, 8, Table S5).

During the drought progression, growth rate of DEX:IPT plants gradually declined, similarly as in WT (Figure S1, Table 1, and Table S2). Leaf water loss at the end of drought was higher than in WT (loss by 30% in comparison with 23% in WT; Figure 2). *AtIPT3* transcription was strongly suppressed and *AtCKX1* transcription was enhanced (Table 2). Transcription of stress-marker genes was high. Drought caused decrease of active CKs by 52% in comparison with activated well-watered control (Figure 3). Nevertheless, tZ highly prevailed. The IAA content decreased (Figure 4). Up-regulation of IAA catabolites – OxIAA and its glucosyl ester was observed (Table S5). ABA content was strongly up-regulated by drought, as well as its metabolites (Figure 5, Table S5). At the end of drought, JA and JA-Ile were diminished, while ACC was moderately increased (Figures 6, 8).

Recovery was associated with fast growth activation, one of the most pronounced among the tested genotypes (comparable with the effect of exogenous CK application; Table 1). At the end of 6-day recovery period, growth rate reached about 215% of the corresponding control. The active CK content was elevated by ca 19%, and IAA content even by ca 75% in comparison with drought conditions (Figures 3, 4). ABA content decreased to the level of induced control, remaining higher than in WT (Figure 5). SA was slightly enhanced in comparison with WT plants (Figure 7).

### CK up-regulation induced during stress progression (SAG:IPT)

Stimulation of *ipt* expression by *SAG12* promoter resulted in substantially different CK dynamics in comparison with DEX:IPT plants. In well-watered plants, no elevation of active CKs was observed 38 DAS (Figure 3). Only tZR and tZR phosphate were increased (by about 300 and 60%, respectively), which may indicate stimulation of *SAG12* promoter activity given by the initiation of the senescence program (Table S4). Significant increase of active CKs (by 46%) was observed at the end of experiment (44 DAS; Figure 3); *AtIPT3* transcription was down-regulated, while *AtCKX1* transcription increased with plant age (Table 2). Simultaneously, moderate decrease of ABA, JA, and SA was found in comparison with 25th DAS (Figures 5–7).

During drought progression, initial drop of growth rate occurred earlier than in DEX:IPT plants; however, in later stages (after 4 days, 29 DAS) relatively higher growth rate was maintained (Figure S1, Table 1, and Table S2). Decrease of water content was the same as in WT (Figure 2). The transcription of stress marker genes was low (Table 2). Under drought stress, SAG:IPT plants exhibited significantly higher F_v_/F_m_ than WT (Figure S2). SAG:IPT was the only genotype, which responded to drought by the increase of active CKs (more than 4-times in comparison with well-watered control). Apart from tZ, also DZ and cZ were elevated (Figure 3). The *AtIPT3* transcription was diminished, while *CKX1* transcription was increased in comparison with the corresponding well-watered control (Table 2). Drought stress resulted in down-regulation of IAA, which, however, remained slightly higher than in the other stressed genotypes (Figure 4). Stress response was associated with strong ABA elevation, the highest among the tested genotypes (Figure 5). Simultaneously, increase of JA and especially of JA-Ile was observed (Figure 6). In the case of ACC mild elevation was also observed in stressed plants (Figure 8).

Recovery was associated with increased growth rate (by 165% of the corresponding well-watered control; recovered SAG:IPT grew by 7% faster than recovered WT; Figure S1, Table 1, and Table S3B). Simultaneously, active CK levels still remained higher than in WT (ca 159% of WT; Figure 3). Up-regulation of active CKs was observed at the end of experiment also in well-watered plants (to ca 182% of WT), probably due to undergoing senescence. The transcription of *AtIPT3* increased to the level of young plants (25 DAS; Table 2). Recovery was associated with IAA elevation, high above the level of the corresponding control (Figure 4). The contents of stress hormones ABA and JA were low (Figures 5, 6).

### CK up-regulation achieved by application of exogenous CK (Col-0 CK)

The exogenous application of aromatic CK *meta*-topolin had minor negative effect on endogenous active CKs (with exception of cZ), which coincided with substantial suppression of *AtIPT3* transcription (Figure 3, Table 2). Under control conditions, IAA synthesis was stimulated, while level of JA, and to minor extent also of JA-Ile, was decreased after abrupt CK up-regulation (Figures 4, 6).

Drought imposed gradual decrease of the growth rate, similar to WT (Figure S1, Table 1, and Table S2). Application of CK resulted in higher water loss than in WT (37 vs. 23% at the end of drought; Figure 2). Drought response of CK treated plants was associated with the decrease of endogenous active CK levels (by 40% in comparison with the well-watered control; Figure 3), CK ribosides (by 32%) and CK phosphates (by 50%; Table S4). The most profound effect was observed in the case of tZ-type and iP-type CKs. Transcription of *AtIPT3* was strongly suppressed (Table 2). Drought stress caused substantial down-regulation of IAA, associated with mild up-regulation of OxIAA (Figure 4, Table S5) and more profound increase of its glucosyl ester (results not shown). ABA was strongly up-regulated by drought, together with its catabolites (mainly phaseic acid and 9′-hydroxy-ABA; Figure 5, Table S5). The other stress hormones, SA, JA and JA-Ile were only slightly elevated (Figures 6, 7), while ACC decreased (Figure 8).

After re-watering, the growth rate of CK treated plants increased fastest of all tested variants (Table 1). At the end of recovery period, the growth rate was by 21% higher than that of WT, reaching about 204% of the corresponding treated control (Table 1, Table S3B). The level of active CKs increased substantially (by ca 20% in comparison with the control and ca 4-times in comparison with the corresponding drought stressed plants). CK phosphates increased more than 4-times in comparison with drought stressed-plants, which correlated well with highly increased *AtIPT3* transcription (Figure 3, Table 2, and Table S4). Recovery was associated with IAA elevation, well above the level in the corresponding control (Figure 4). Recovery led to reduction of ABA, JA, and JA-Ile contents (Figures 5, 6).

#### The impact modulated cytokinin content on the other hormones

Modulation of the content of one hormone usually results in changes of the other hormone levels, in order to keep the desirable hormone ratio. Close relationship exists between CKs and auxins. Both CKs and auxins are indispensable for cell cycle progression and thus for cell division (Laureys et al., 1998). They are necessary for coordination of the growth of the above- and under-ground parts of the plant. Their ratio controls morphology of growing tissues (Miller et al., 1955). Under control conditions, CK suppression by *CKX* overexpression was accompanied by decrease of IAA (Figure 4). This feature was evident especially in case of long-term CK down-regulation in 35S:CKX transformant. Accordingly, the application of exogenous CK promoted IAA formation.

Another hormone, which exhibits an intensive cross-talk with CKs, is ABA. CK/ABA ratio affects stomata aperture (e.g., Skalák et al., 2016), which is crucial for regulation of the water loss, but also for the supply of carbon dioxide for photosynthesis. In well-watered conditions, the lowest ABA content was determined in 35S:CKX plants (decrease of ABA by ca 53% in comparison with WT correlated well with decrease of active CKs by ca 50%; Figure 5).

### Multivariate analyses highlight similarities in observed patterns

**Figure 9:**
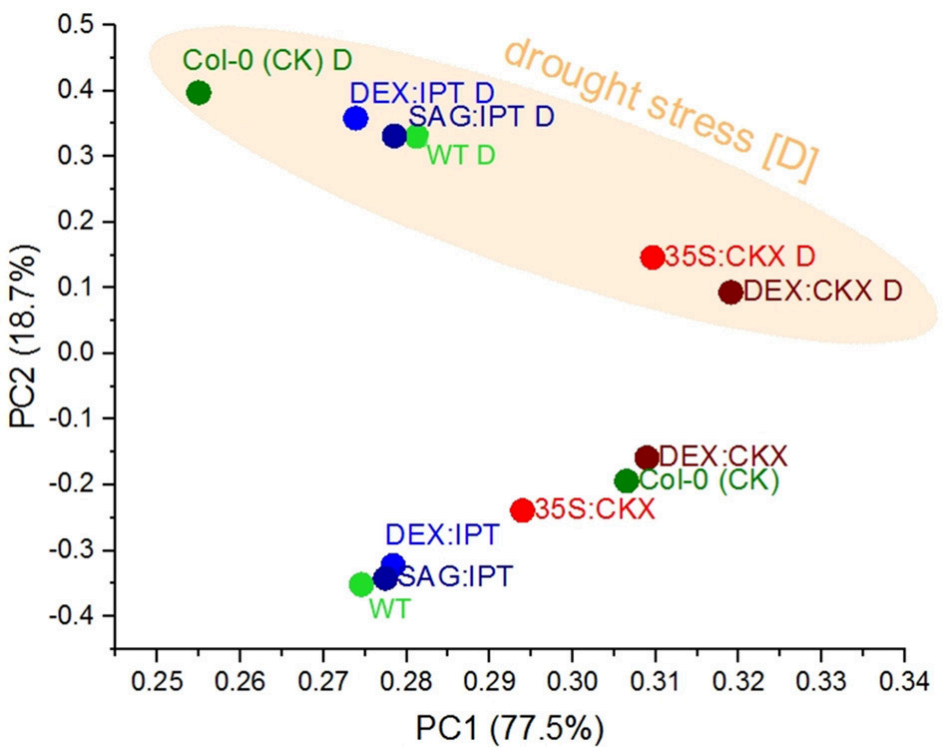
Effects of drought and cytokinin pool size on growth rates of *Arabidopsis* plants. The growth rates of individual *Arabidopsis* lines recorded during the drought stress and recovery period and of their respective controls were analyzed by principal component analysis (PCA). The PCA clearly separates the drought stressed plants from the control groups along the PC2, and PC1 illustrates a similarity in the growth rate dynamics between lines with the constitutive and inducible CKX expression.

We performed a set of multivariate analyses to identify similarities in observed patterns. The hierarchical clustering and PCA analyses of leaf area dynamics showed a clear separation of stressed plants from their respective controls with the exception being 35S and DEX-inducible *CKX* overexpresser lines, which exhibited relatively minor stress impact. Analyses also showed that the total leaf areas of DEX:IPT and Col-0 CK plants had the best pattern match and their close similarity is reflected also in the drought:control ratios of leaf area and growth rates (data not shown). The most informative proved to be the PCA of growth rates (Figure 9) that separates drought stress along the PC2 and individual genotypes/treatments along the PC1.

**Figure 10:**
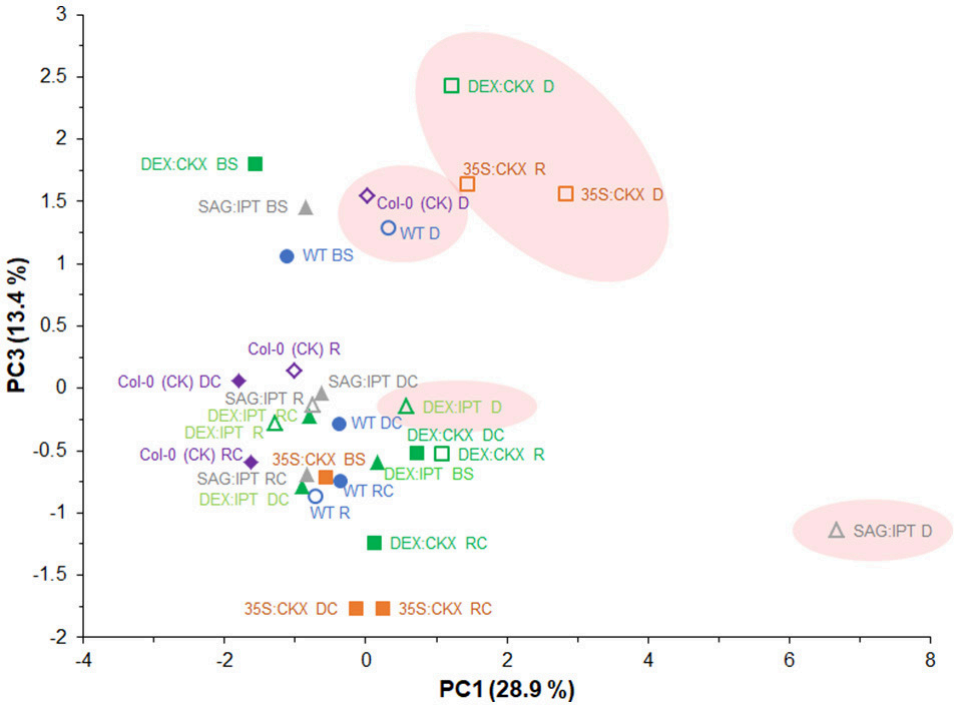
Effects of drought and modulation of cytokinin metabolism on the levels of active hormones (active CKs, IAA, ABA, JA, SA and ACC) in *Arabidopsis* plants. The hormone levels in individual variants determined before stress (BS), during the drought stress (D) and recovery period (R) and of their respective controls (DC, RC) were analyzed by principal component analysis (PCA). PC1 illustrates a link among the stress hormones ABA, JA, ACC and cZ as well as dihydrozeatin. Separation along PC3 clusters stressed and non-stressed plants according to the content of stress hormones ABA, JA, SA and ACC, showing distinct behavior of SAG:IPT and DEX:IPT.

PCA analysis of active hormones (namely active CKs, IAA, ABA, JA, SA, and ACC; Figure 10) revealed distinct response of drought-stressed SAG:IPT plants, which enhanced CK levels during stress progression (PC1). The clustering of stressed DEX:IPT plants with well-watered plants of all genotypes showed the effect of enhanced CK levels on delay in activation of stress defense, which may diminish drought impact (at least in the stress strength used in our experiment). Distribution along PC1 illustrates a link between the stress hormones and low active CKs - cZ and dihydrozeatin. Separation along PC3 demonstrated the importance of the stress hormones, namely of ABA, JA, ACC, and SA in drought response. Clustering of 35S:CKX plants in drought stress and after rewatering reflected maintenance of enhanced defense mechanisms in this genotype also after the stress release (at least for some period).

**Figure 11:**
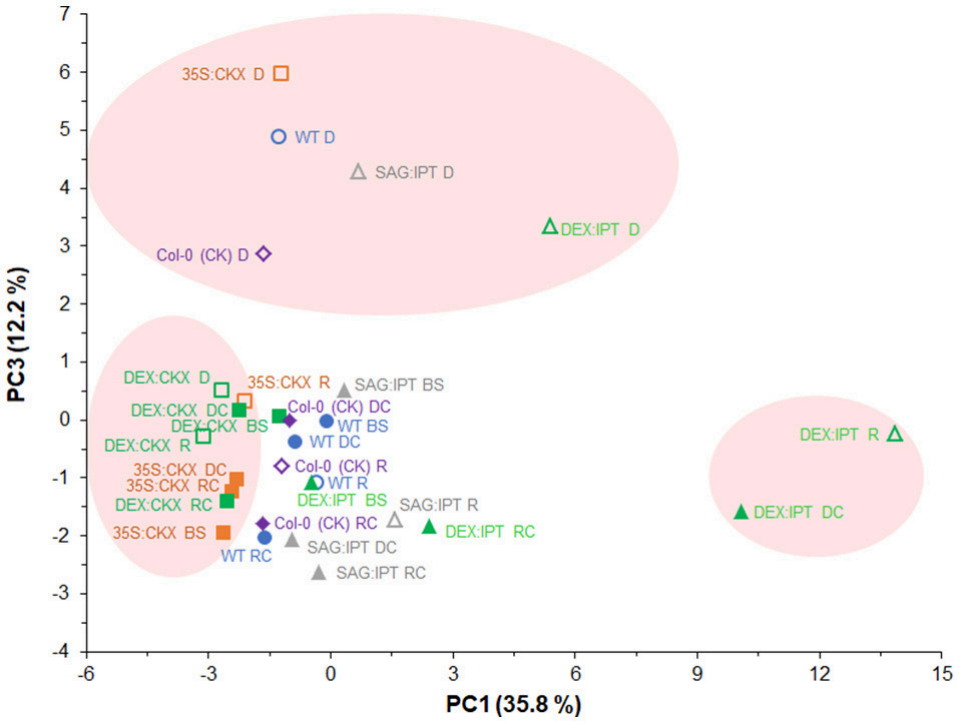
Effects of drought and modulation of cytokinin metabolism on the content of all determined hormone-related metabolites in *Arabidopsis* plants. Metabolite profiling recorded before stress (BS), during the drought stress (D) and recovery period (R) and of their respective controls (DC, RC) were analyzed by principal component analysis (PCA). PC1 clustered variants according to CKs content. The PCA confirmed the importance of ABA (and its metabolites) and of JA/JA-Ile in drought response of all experimental variants (PC3). Clustering of DEX:IPT plants after re-watering (R) and of drought-stressed control (DC) along the PC1 indicates positive effect of CKs on the recovery.

PCA analysis of all determined hormone-related metabolites (Figure 11) showed clear separation of tested genotypes according to CK content along the PC1. Separation along PC3 confirmed the importance of ABA (and its metabolites) and of JA/JA-Ile in drought response of all experimental variants. Clustering of DEX:CKX plants close to its well-watered control with strongly stimulated CK degradation indicates that down-regulation of active CKs is an inherent stress response. Clustering of DEX:IPT plants after re-watering (R) and of drought-stressed control (DC) indicates positive effect of CKs in recovery.

## Discussion

### Tendency of transgenic plants to re-establish hormonal homeostasis

#### Regulation of endogenous cytokinin levels

Modification of phytohormone levels in plants is usually achieved by overexpression of the genes for biosynthetic or degradation enzymes (Gan and Amasino, 1995; Werner et al., 2001). However, the final effect on hormonal pool depends not only on the activity of the introduced genes but also on the plant response to the disturbed hormonal homeostasis (Figures 10, 11).

In activated DEX:CKX plants, the overexpression of CK catalytic enzyme *HvCKX2* led to suppression of endogenous *AtCKX1* expression and up-regulation of expression of CK biosynthetic gene *AtIPT3* (Table 2). Thus, expression of CK biosynthetic gene(s) was promoted by intensive CK degradation, in order to diminish CK imbalance. Low levels of CK precursors seemed to indicate high CK turn-over due to the fast degradation rather than down-regulated CK biosynthesis (Table S4). Simultaneously, the other deactivation pathways (CK N- and O-glucosylation) were down-regulated, which might suggest tendency to maintain CK homeostasis as much as possible (Table S4).

Accordingly, overexpression of *ipt* in DEX:IPT or SAG:IPT genotypes led to the inhibition of endogenous *AtIPT3* transcription and in the case of SAG:IPT also to stimulation of expression of endogenous *AtCKX1* (Table 2). In both genotypes O-glucosylation pathway was promoted (Table S4). Similar effects as *ipt* expression were imposed by application of exogenous CK *meta*-topolin. The modulation of CK metabolism had significant effect on the resulting levels of active CKs in all genotypes.

Apart from the introduced gene, important role is played by the promoter (Figures 9–11). Constitutive *AtCKX1* expression under 35S promoter strongly decreased the growth rate of the transformed plants (Figure S1, Table 1), having also significant impact on plant morphology (enhanced root growth, thicker and darker leaves; Werner et al., 2001). Use of dexamethasone-inducible promoter allowed distinguishing of the impact of slow growth from the morphological effects. The *ipt* induction by dexamethasone at the drought stress onset (supported by two stimulations during the stress progression) and stress-induced *ipt* expression under *SAG12* promoter enabled to compare the impact of the timing of CK elevation. Our data indicated better performance of the transformant with the construct containing senescence-inducible promoter *SAG12* than the dexamethasone-inducible one.

### Plant responses to drought

In all tested genotypes, drought response was associated with suppression of growth, accompanied with down-regulation of active CK levels (especially of tZ), even in transformants with strongly stimulated *ipt* gene expression (Figures 3, 9–11, Figure S1, Table 1, and Table S2). This is in accordance with other reports on different species, e.g., tomato (Kudoyarova et al., 2007). The only exception in CK regulation was SAG:IPT genotype, due to the gradual activation of *SAG12* promoter by drought-strengthened senescence (Vanková et al., 2012).

During drought progression, all genotypes gradually diminished their growth rate (Figure S1, Table 1). Metabolism of 35S:CKX plants was adjusted to slow growth even under control conditions, which was accompanied by enhanced basal level of the stress associated transcripts (e.g., *AtP5CS1*; Table 2). These results evidence high “preparedness” of 35S:CKX transformants for the unfavorable conditions (Figure 9). 35S:CKX plants were not affected by mild stress; however, they responded very effectively to severe drought (fast and strong decrease of the growth rate – ca 32% of stressed WT, as well as strong increase of ABA levels; Figure 5, Figure S1, Table S2). Dexamethasone-induced stimulation of *HvCKX2* substantially down-regulated the growth rate, both under control and stress conditions (Figure 9). After the initial drop caused by *CKX* activation, the growth rate inhibition followed the same trend as in WT (at the level of ca 75% of stressed WT; Table S2). Sudden decrease of active CKs at the stress onset might contribute to stomata closure, as this variant preserved the water content best from the studied genotypes.

Stimulation of *ipt* with dexamethasone slightly promoted plant growth under control conditions (Figure S1, Table S2). However, in drought, the growth rate was suppressed even faster than in WT (Table 1). Mild increase of ABA was found in DEX:IPT plants, in comparison with WT (by ca 10%; Figure 5), probably in order to balance, at least partially, the elevated CK content. Nevertheless, CK/ABA ratio was still enhanced (ca 0.15 vs. ca 0.03), which might be the reason of higher water loss in this genotype in comparison with WT and especially with CKX overexpressers. Similar negative effect on water relations was observed also after application of exogenous CK *meta*-topolin.

SAG:IPT plants were the only genotype, which exhibited elevation of the active CK content during drought progression (in comparison with non-stressed control more than 4-times; Figure 3). This increase was given by promotion of *SAG12* promoter activity by drought, which was shown in detail in tobacco (Vanková et al., 2012). Stimulation of *ipt* expression led to elevation of active CKs as well as of iPR phosphate and cZ riboside O-glucoside together with down-regulation of *AtIPT3* and up-regulation of *AtCKX1* transcription (Figure 3, Table 2, and Table S4). The CK dynamics of SAG:IPT differed from the dynamics of DEX:IPT. No sudden CK increase took place in SAG:IPT plants, which might be, together with the most profound ABA elevation (Figure 5), the reason of the lower water loss. In the case of SAG:IPT, the up-regulation of CKs occurred also in well-watered plants at the end of the experiment (Figure 3), probably due to the promoter activation by the onset of natural senescence.

In spite of the fact that JA is the key hormone in the defense to necrotroph or herbivore attack, the increasing evidence has shown that it plays an important role also in drought response (Djilianov et al., 2013), which was confirmed by our PCAs (Figures 10, 11). Increase of JA and/or JA-Ile (to higher or lower extent) was observed in all genotypes with exception of DEX:IPT plants, which might reflect antagonistic relationship between JA and CKs in regulation of senescence program, chlorophyll content and cell division (e.g., Liu et al., 2016; Figure 6).

### Plant response during the recovery phase (after re-watering)

Recovery was associated with vigorous growth, accompanied by high elevation of active CKs (Figure 3). Within 1 day after re-watering, growth rates of all variants accelerated and exceeded those of corresponding well-watered plants, with the exception of both CKX overexpressers (Figure S1, Table 1). These genotypes with down-regulated CK levels (predominantly 35S:CKX) maintained elevated ABA levels and exhibited high levels of JA as well as of JA-Ile (higher than controls; Figures 5, 6). PCA analyses (Figures 10, 11) indicated that CKX plants (especially 35S:CKX) remained (at least for some time) prepared for another stress period.

In contrast, the fastest recovery and the highest growth rate reached in the end of recovery period were found in the variant sprayed with *meta*-topolin and in DEX:IPT plants (Figure 9, Figure S1, and Table 1). These lines with up-regulated CKs (especially DEX:IPT) enhanced predominantly tZ-type CKs, including tZ–the most physiologically active CK in the stimulation of cell division (Figure 3, Table S4). Similar response was exhibited also by WT. The plants treated with exogenous CK maintained significantly higher SA levels than the corresponding controls (Figure 7). Together with DEX:IPT, they strongly down-regulated JA content during recovery (Figure 6).

Both *ipt* transformants maintained high expression level of the introduced gene also during the recovery period, which was associated with very high content of CK phosphates (mainly tZR phosphate) as well as of CK transport form tZR (Tables 2, S4). Simultaneously, CK deactivation pathways were active, which indicates tight regulation of CK homeostasis.

## Conclusions

The possibility to enhance plant drought tolerance by modulation of CK levels depends on the stress duration, severity (soil water potential) and speed of dehydration. The actual conditions substantially affect performance of the transformants.

Various determined parameters, e.g., shoot area expansion rate, indicated that the most drought tolerant genotype was 35S:CKX line. In this case, both low CK levels and permanent adaptation to slow growth (plant morphology, up-regulated basal transcription of stress-related genes, e.g., *P5CS1*) contributed to the stress tolerance. Constant up-regulation of defense (before and after stress period) indicated better “preparedness” of this CKX genotype to stress. Dwarf shoot phenotype, however, is not in favor of potential application in agriculture.

Dexamethasone-induced down-regulation of active CKs resulted in the decrease of the growth rate. Transient character of *CKX* overexpression was associated with earlier switch-off of defense mechanisms after stress release, in comparison with 35S:CKX plants. However, sudden CK decrease after *HvCKX2* stimulation had a significant positive effect on plant water relations, probably due to the promotion of stomata closure.

Up-regulation of CK levels at the stress onset (repeated twice during the stress progression) by induction of *ipt* expression or by application of stable aromatic CK *meta*-topolin, had highly significant positive effect on plant recovery after rehydration, diminishing the stress effects. However, CK elevation was associated with higher water loss, which might be disadvantageous in case of severe long-term stress.

The up-regulation of CKs driven by senescence inducible promoter (SAG:IPT plants) led to slower recovery in comparison with DEX:IPT plants (comparable with WT). However, the water loss was substantially diminished. SAG:IPT transformant has been shown to be the most advantageous at least in conditions of relatively short-term drought stress.

Our results illustrate different plant strategies to cope with drought and demonstrate that CKs play an important role in both stimulatory and “quiescent” plant approach.

## Author contributions

RV, FF, NK, US, and SP contributed conception and design of this study; RV, RP, US, and FF arranged for financial support; BB, MČ, LS, and JH provided Arabidopsis seeds and information about plant physiology; SP, NK, FF, and JH performed the experiment; VK, PD, AG, SP, and NK analyzed samples; RV, SP, FF, MČ, and TV evaluated results; RV, SP, and FF wrote the manuscript. All authors contributed to manuscript revision, read and approved the submitted version.

### Conflict of interest statement

The authors declare that the research was conducted in the absence of any commercial or financial relationships that could be construed as a potential conflict of interest.

## Abbreviations

35S:CKX: *AtCKX1* gene under constitutive promoter *35S*
ABA: abscisic acid
ACC: aminocyclopropane carboxylic acid
CK: cytokinin
CKX: cytokinin oxidase/dehydrogenase
Col-0 CK: *Arabidopsis thaliana* plants sprayed with exogenous cytokinin
cZ: *cis*-zeatin
DAS: days after sowing
DEX:IPT: *ipt* gene under dexamethasone-inducible promoter
DEX:CKX: *HvCKX2* gene under dexamethasone-inducible promoter
DZ: dihydrozeatin
IAA: indole-3-acetic acid
iP: isopentenyladenine
iPR: isopentenyladenosine
IPT: isopentenyl transferase
JA: jasmonic acid
JA-Ile: jasmonate-isoleucine
NCED3: 9-cis-epoxycarotenoid dioxygenase 3
OxIAA: 2-oxindole-3-acetic acid
P5CS1: δ-1-pyrroline-5-carboxylate synthase 1
RD29B: responsive to desiccation 29B
SA: salicylic acid
SAG: senescence-associated gene
SAG:IPT: *ipt* gene under senescence-inducible promoter *SAG12*
SARK: senescence-associated receptor-like kinase
tZ: *trans*-zeatin
tZR: *trans*-zeatin riboside
WT: wild-type.
