# Supplementary material for: Cytokinins: Their Impact on Molecular and Growth Responses to Drought Stress and Recovery in Arabidopsis
Source: Front Plant Sci. 2018 May 22;9:655. doi: 10.3389/fpls.2018.00655 (PMC5972670; doi:10.3389/fpls.2018.00655)

Cytokinins: their impact on molecular and growth responses to drought stress and recovery in *Arabidopsis*

Sylva Prerostova, Petre I. Dobrev, Alena Gaudinova, Vojtech Knirsch, Niklas Körber, Roland Pieruschka, Fabio Fiorani, Bretislav Brzobohaty, Martin, Cerny, Lukas Spichal, Jan Humplik, Tomas Vanek, Ulrich Schurr, Radomira Vankova

Table S1. The list of primers used in RT-qPCR.

Gene	Forward	Reverse
AtUBQ10 (At4g05320)	gaagttcaatgtttcgtttcatgt	ggattatacaaggccccaaaa
HvCKX2 (AF490591)	aacgctcactgaagggaa	attctcggggcagcaag
ipt from *Agrobacterium*	atcctccctcaagaataagc	ctgaaaggaacgacgc
AtCKX1 (At2g41510)	cacctttggcaattctacat	tgtccttgaagcgagtga
AtIPT3 (At3g63110)	ttttcaggaacgagcagt	ttggaccttcgctttgta
AtRD29B (At5g52300)	caggatcaacggtgatgaca	tgcgtctccttcactccac
AtP5CS1 (At2g39800)	aacgccagcacaagattc	cctctcattatccatctcgttgt
AtNCED3 (At3g14440)	gacgagaatctcaagagtg	ccgagcatgtttctgttgac

Table S2. Daily growth rates of all experimental variants. Growth rate of each plant was calculated from rosette area in mm^2 determined by RGB measurement. Mean growth rate ± SD was calculated from growth rates of each variant in corresponding time (n = 15-40). Measurement was performed before stress (22-25 DAS), during drought (28-38 DAS) and during recovery (39-44 DAS). Statistically significant differences (p < 0.01, two-sample t-test) between the corresponding variants (drought or control) of WT and transformants within each time-point are marked by *; and between stressed and control variants within each genotype in each time-point are marked in bold.

DAS	WT		35S:CKX		DEX:CKX	
	control	stressed	control	stressed	control	stressed
22	0.248 ± 0.017	0.247 ± 0.019	0.124 ± 0.023*	0.122 ± 0.016*	0.245 ± 0.022	0.247 ± 0.018
23	0.245 ± 0.047	0.236 ± 0.028	0.141 ± 0.026*	0.133 ± 0.033*	0.243 ± 0.038	0.238 ± 0.035
24	0.227 ± 0.029	0.213 ± 0.027	0.132 ± 0.025*	0.128 ± 0.033*	0.228 ± 0.026	0.213 ± 0.035
25	0.212 ± 0.032	0.225 ± 0.025	0.125 ± 0.026*	0.120 ± 0.033*	0.225 ± 0.032	0.234 ± 0.029
28	0.183 ± 0.043	0.178 ± 0.030	0.089 ± 0.023*	0.091 ± 0.031*	0.139 ± 0.023*	0.130 ± 0.021*
29	0.193 ± 0.036	**0.159 ± 0.036**	0.089 ± 0.022*	**0.064 ± 0.028***	0.137 ± 0.030*	0.123 ± 0.031*
30	0.182 ± 0.028	**0.130 ± 0.032**	0.091 ± 0.027*	**0.046 ± 0.016***	0.152 ± 0.024*	**0.120 ± 0.022**
31	0.145 ± 0.034	0.148 ± 0.038	0.075 ± 0.025*	**0.050 ± 0.024***	0.105 ± 0.029*	0.105 ± 0.025*
32	0.179 ± 0.036	**0.111 ± 0.028**	0.067 ± 0.024*	**0.034 ± 0.019***	0.118 ± 0.027*	**0.081 ± 0.021***
35	0.152 ± 0.035	**0.086 ± 0.032**	0.070 ± 0.023*	**0.012 ± 0.016***	0.109 ± 0.018*	**0.063 ± 0.023***
36	0.130 ± 0.030	**0.035 ± 0.026**	0.038 ± 0.022*	**0.003 ± 0.017***	0.067 ± 0.024*	**0.046 ± 0.022**
37	0.194 ± 0.032	**0.037 ± 0.026**	0.085 ± 0.016*	**0.009 ± 0.019***	0.114 ± 0.025*	**0.026 ± 0.026**
38	0.138 ± 0.031	**0.020 ± 0.029**	0.082 ± 0.016*	**0.001 ± 0.015***	0.120 ± 0.024*	**0.016 ± 0.026**
39	0.124 ± 0.024	0.143 ± 0.033	0.059 ± 0.012*	**0.005 ± 0.032***	0.091 ± 0.023*	**0.053 ± 0.027***
42	0.118 ± 0.034	**0.227 ± 0.024**	0.059 ± 0.017*	**0.083 ± 0.018***	0.097 ± 0.018	**0.125 ± 0.024***
44	0.083 ± 0.013	**0.148 ± 0.022**	0.036 ± 0.019*	0.049 ± 0.016*	0.069 ± 0.027	0.085 ± 0.024*

DAS	DEX:IPT		SAG:IPT		Col-0 CK	
	control	stressed	control	stressed	control	stressed
22	0.241 ± 0.022	0.242 ± 0.021	0.246 ± 0.025	0.248 ± 0.013	0.248 ± 0.019	0.239 ± 0.021
23	0.236 ± 0.035	0.249 ± 0.037	0.255 ± 0.057	0.247 ± 0.023	0.241 ± 0.037	0.236 ± 0.034
24	0.218 ± 0.028	0.218 ± 0.027	0.220 ± 0.025	0.224 ± 0.026	0.224 ± 0.024	**0.205 ± 0.027**
25	0.234 ± 0.038	0.216 ± 0.032	0.234 ± 0.037	0.234 ± 0.029	0.227 ± 0.029	0.216 ± 0.029
28	0.186 ± 0.035	0.168 ± 0.029	0.196 ± 0.032	**0.173 ± 0.019**	0.164 ± 0.034	0.147 ± 0.042*
29	0.220 ± 0.039*	**0.144 ± 0.031**	0.205 ± 0.030	**0.166 ± 0.019**	0.208 ± 0.044	**0.115 ± 0.063***
30	0.200 ± 0.029	**0.128 ± 0.026**	0.189 ± 0.030	**0.165 ± 0.026***	0.166 ± 0.034	**0.113 ± 0.042**
31	0.155 ± 0.029	**0.130 ± 0.044**	0.158 ± 0.028	0.140 ± 0.035	0.173 ± 0.034*	**0.121 ± 0.048**
32	0.175 ± 0.037	**0.071 ± 0.021***	0.186 ± 0.030	**0.111 ± 0.028**	0.141 ± 0.032*	**0.074 ± 0.034***
35	0.137 ± 0.029	**0.045 ± 0.020***	0.150 ± 0.034	**0.077 ± 0.030**	0.129 ± 0.028*	**0.064 ± 0.028***
36	0.139 ± 0.028	**0.020 ± 0.027**	0.140 ± 0.027	**0.033 ± 0.027**	0.116 ± 0.031	**0.025 ± 0.028**
37	0.177 ± 0.034	**0.017 ± 0.027***	0.205 ± 0.040	**0.028 ± 0.027**	0.153 ± 0.043*	**0.007 ± 0.041***
38	0.134 ± 0.038	**0.017 ± 0.027**	0.150 ± 0.039	**0.013 ± 0.036**	0.124 ± 0.034	**0.019 ± 0.031**
39	0.130 ± 0.039	0.152 ± 0.066	0.136 ± 0.031	0.117 ± 0.041	0.126 ± 0.038	**0.185 ± 0.044***
42	0.119 ± 0.035	**0.209 ± 0.037**	0.133 ± 0.028	**0.252 ± 0.033**	0.129 ± 0.041	**0.200 ± 0.048**
44	0.080 ± 0.025	**0.173 ± 0.036**	0.096 ± 0.026	**0.158 ± 0.021**	0.088 ± 0.030	**0.179 ± 0.043**

Table S3. A) Ratio of mean growth rate of non-stressed transformants and mean growth rate of non-stressed WT (expressed in %). B) Ratio of mean growth rate of stressed genotype and mean growth rate of stressed WT (in %). The individual mean growth rates are shown in Table S2.

A)

DAS	35S:CKX	DEX:CKX	DEX:IPT	SAG:IPT	Col-0 CK	
**22**	50.1	98.8	97.5	99.5	100.0	
**23**	57.5	99.1	96.4	104.4	98.5	
**24**	58.0	100.7	96.1	97.2	99.0	
**25**	58.9	106.0	110.1	110.3	106.9	
**28**	48.9	76.3	101.7	107.1	89.6	drought
**29**	46.0	70.9	113.8	106.4	107.8	
**30**	50.0	83.7	109.9	104.0	91.5	
**31**	51.6	72.1	107.0	108.8	119.1	
**32**	37.3	65.8	98.3	104.3	79.2	
**35**	46.0	72.1	90.5	98.7	85.2	
**36**	29.3	51.7	106.7	107.2	89.5	
**37**	43.8	58.7	91.5	105.5	79.1	
**38**	59.3	86.7	96.8	108.5	90.0	
**39**	47.3	73.3	105.2	109.6	101.5	re-watering
**42**	49.8	82.5	101.0	112.2	109.2	
**44**	42.8	82.7	96.9	115.6	105.7	

B)

DAS	35S:CKX	DEX:CKX	DEX:IPT	SAG:IPT	Col-0 CK	
**22**	49.3	100.2	98.2	100.4	96.7	
**23**	56.4	100.7	105.6	104.6	100.0	
**24**	60.3	99.9	102.3	105.2	96.5	
**25**	53.3	103.9	96.0	103.7	96.0	
**28**	51.3	72.7	94.1	97.2	82.6	drought
**29**	40.6	77.4	90.6	104.4	72.4	
**30**	35.3	92.2	98.1	126.4	86.8	
**31**	33.5	70.8	87.3	94.2	81.3	
**32**	30.5	72.6	63.8	99.8	66.4	
**35**	13.8	72.6	52.1	89.1	73.8	
**36**	9.3	72.4	56.9	93.5	70.5	
**37**	24.7	70.4	45.9	73.8	18.8	
**38**	4.3	78.3	85.6	62.4	91.5	
**39**	3.7	36.9	106.7	81.9	129.6	re-watering
**42**	36.7	54.9	92.0	111.0	88.0	
**44**	33.4	57.6	117.2	107.0	121.2	

Table S4. The impact of drought stress and recovery on cytokinin (CK) metabolites in different genotypes. CK Ribosides: *trans*-zeatin riboside, dihydrozeatin riboside, isopentenyladenosine, *cis*-zeatin riboside. CK Phosphates: *trans*-zeatin riboside phosphate, dihydrozeatin riboside phosphate, isopentenyladenosine phosphate, *cis*-zeatin riboside phosphate. CK N-glucosides: *trans*-zeatin-7-glucoside, *trans*-zeatin-9-glucoside, dihydrozeatin-7-glucoside, dihydrozeatin-9-glucoside, isopentenyladenine-7-glucoside, isopentenyladenine-9-glucoside, *cis*-zeatin-9-glucoside. CK O-glucosides: *trans*-zeatin-O-glucoside, *trans*-zeatin riboside-O-glucoside, dihydrozeatin riboside-O-glucoside, *cis*-zeatin riboside-O-glucoside. Values give mean concentrations of phytohormones in pmol/g DW ± SD (n = 6). Statistically significant ($p < 0.05$, two-sample t-test) differences between the corresponding variants of WT and transformant are marked by *, and between D/DC or R/RC variants within each genotype in bold.

		**CK Ribosides**	**CK Phosphates**	**CK N-glucosides**	**CK O-glucosides**
**WT**	**before stress**	274.8 ± 18.5	25.2 ± 7.3	1810.3 ± 411.5	122.0 ± 49.9
**WT**	**drought control**	241.3 ± 39.5	19.8 ± 2.8	1342.1 ± 281.6	161.7 ± 65.9
**WT**	**drought**	**90.9 ± 11.7**	**7.4 ± 2.2**	1087.8 ± 156.3	156.5 ± 32.1
**WT**	**recovery control**	201.2 ± 50.3	20.0 ± 6.0	644.7 ± 193.4	89.9 ± 36.0
**WT**	**recovery**	296.1 ± 59.5	**36.0 ± 7.2**	**1400.9 ± 131.7**	165.1 ± 71.5
**35S:CKX**	**before stress**	38.2 ± 12.9*	12.7 ± 1.4*	102.3 ± 25.1*	38.2 ± 15.37*
**35S:CKX**	**drought control**	37.7 ± 20.7*	10.3 ± 3.1*	116.8 ± 60.2*	77.4 ± 42.9
**35S:CKX**	**drought**	41.0 ± 9.5*	9.0 ± 3.6	140.5 ± 28.3*	**204.8 ± 36.7**
**35S:CKX**	**recovery control**	66.5 ± 12.8*	10.7 ± 1.5*	131.1 ± 16.9*	100.2 ± 17.5
**35S:CKX**	**recovery**	71.9 ± 12.8*	**17.1 ± 4.0***	125.2 ± 12.5*	84.0 ± 24.9
**DEX:CKX**	**before stress**	56.1 ± 14.0*	14.6 ± 4.4	372.9 ± 111.9*	194.9 ± 78.0
**DEX:CKX**	**drought control**	10.3 ± 2.6*	3.0 ± 0.9*	360.5 ± 17.3*	134.0 ± 44.5
**DEX:CKX**	**drought**	7.4 ± 1.8*	1.8 ± 0.6*	**239.9 ± 65.7***	**86.4 ± 3.0***
**DEX:CKX**	**recovery control**	79.2 ± 2.9*	16.1 ± 1.6	362.5 ± 11.1*	96.0 ± 5.3
**DEX:CKX**	**recovery**	**31.1 ± 1.4***	**10.5 ± 1.4***	**205.5 ± 15.9***	**49.3 ± 4.0***
**DEX:IPT**	**before stress**	251.3 ± 10.7	18.4 ± 5.5	1425.6 ± 307.3	125.5 ± 56.2
**DEX:IPT**	**drought control**	3356.8 ± 839.2*	191.2 ± 57.4*	10155.3 ± 3046.6*	1866.0 ± 746.4*
**DEX:IPT**	**drought**	**1523.6 ± 380.9***	84.9 ± 25.5*	7319.6 ± 2195.9*	1568.3 ± 627.3*
**DEX:IPT**	**recovery control**	856.9 ± 214.2*	91.2 ± 27.4*	4167.4 ± 1250.2*	740.8 ± 296.3*
**DEX:IPT**	**recovery**	**2786.2 ± 696.6***	300.0 ± 90.0*	17075.0 ± 5122.5*	3198.9 ± 1279.6*
**SAG:IPT**	**before stress**	295.9 ± 74.0	6.4 ± 1.9*	2237.8 ± 671.4	111.3 ± 44.5
**SAG:IPT**	**drought control**	361.9 ± 90.5	29.0 ± 1.3*	1374.1 ± 412.2	197.5 ± 38.9
**SAG:IPT**	**drought**	345.8 ± 23.1*	**89.4 ± 26.8***	1506.6 ± 124.5*	**623.7 ± 53.2***
**SAG:IPT**	**recovery control**	641.7 ± 18.7*	79.7 ± 3.9*	1250.3 ± 69.2*	321.2 ± 9.8*
**SAG:IPT**	**recovery**	**1006.6 ± 21.1***	**175.3 ± 9.8***	**1984.2 ± 595.3**	**497.2 ± 37.3***
**WT**	**before stress**	274.8 ± 18.5	25.2 ± 7.3	1810.3 ± 411.5	122.0 ± 49.9
**Col-0 CK**	**drought control**	50.4 ± 2.6*	6.3 ± 0.2*	310.4 ± 16.5*	143.6 ± 29.1
**Col-0 CK**	**drought**	**34.4 ± 1.8***	**3.2 ± 0.3***	**404.1 ± 52.6***	110.1 ± 19.7
**Col-0 CK**	**recovery control**	175.9 ± 17.3	29.2 ± 5.5	397.6 ± 39.5*	79.8 ± 5.8
**Col-0 CK**	**recovery**	194.3 ± 8.1*	**14.8 ± 1.1***	**519.4 ± 10.1***	**133.9 ± 19.2**

Table S5. Auxin and abscisic acid (ABA) metabolites. OxIAA – 2-oxindole-3-acetic acid; PAA – phenylacetic acid; DPA – dihydrophaseic acid; PA – phaseic acid; 9OH-ABA – 9´-hydroxy-ABA. Values give mean concentrations of phytohormones in pmol/g DW ± SD (n = 6). Before stress application – BS (25 DAS); well-watered plants at the end of drought period – DC (38 DAS); drought stressed plants – D (38 DAS); well-watered plants at the end of recovery period – RC (44 DAS); re-watered plants – R (44 DAS). Values give mean concentrations of phytohormones in pmol/g DW ± SD (n = 6). Statistically significant ($p < 0.05$, two-sample t-test) differences between the corresponding variants of WT and transformant are marked by *, and between D/DC or R/RC variants within each genotype in bold.

		OxIAA	PAA	DPA	PA	9OH-ABA
WT	BS	3719.6 ± 1859.8	4578.4 ± 667.4	1478.5 ± 198.6	261.9 ± 65.5	192.3 ± 57.7
WT	DC	4370.1 ± 2501.1	4236.1 ± 804.0	1109.5 ± 100.5	355.6 ± 131.6	159.3 ± 47.8
WT	D	5790.7 ± 2895.3	3572.1 ± 1071.9	**1573.2 ± 216.7**	**817.6 ± 37.9**	**293.8 ± 74.2**
WT	RC	4257.8 ± 2128.9	1648.4 ± 576.9	709.0 ± 148.9	347.2 ± 72.9	113.7 ± 60.5
WT	R	8555.7 ± 4277.8	2191.5 ± 724.5	**1834.2 ± 904.0**	378.0 ± 85.0	106.6 ± 21.8
35S:CKX	BS	5101.1 ± 2550.6	5130.8 ± 382.7	661.4 ± 138.9*	76.0 ± 18.4*	32.7 ± 9.8*
35S:CKX	DC	11408.6 ± 5704.3	4412.1 ± 783.3	812.9 ± 359.3	169.7 ± 35.4*	46.3 ± 5.1*
35S:CKX	D	18532.0 ± 9266.0*	3989.5 ± 816.7	**2100.3 ± 879.8**	**1187.8 ± 249.4***	**287.7 ± 119.1**
35S:CKX	RC	13642.6 ± 6821.3*	2026.9 ± 709.4	595.9 ± 125.1	200.6 ± 96.9	34.8 ± 10.4*
35S:CKX	R	19837.7 ± 9918.9	4197.1 ± 1958.4	1026.2 ± 334.5	146.4 ± 32.2*	53.7 ± 16.7*
DEX:CKX	BS	1108.3 ± 554.2*	4333.9 ± 1201.9	1461.4 ± 306.9	114.5 ± 24.1*	128.3 ± 38.5
DEX:CKX	DC	4081.8 ± 902.5	8346.8 ± 2921.4*	1399.1 ± 103.9*	201.1 ± 19.2	102.7 ± 19.9
DEX:CKX	D	2449.8 ± 1182.9	**3053.6 ± 960.1**	**971.3 ± 171.4***	306.4 ± 103.8*	164.1 ± 69.4
DEX:CKX	RC	2225.9 ± 1112.9	906.2 ± 267.9	422.2 ± 13.7*	145.7 ± 24.5*	46.5 ± 1.2*
DEX:CKX	R	1623.1 ± 619.6*	831.2 ± 202.0*	**670.7 ± 13.0***	**99.1 ± 3.0***	41.7 ± 1.3*
DEX:IPT	BS	3004.1 ± 1502.1	3993.1 ± 4897.6	1176.7 ± 261.2	111.2 ± 53.9*	92.1 ± 27.6*
DEX:IPT	DC	7115.5 ± 2878.3	3335.5 ± 973.9	1341.5 ± 272.3	345.8 ± 135.5	148.9 ± 44.7
DEX:IPT	D	10115.7 ± 3981.2	2569.7 ± 716.4	2398.5 ± 894.4	**1117.3 ± 110.2***	**338.2 ± 65.5**
DEX:IPT	RC	8822.4 ± 5586.4	2623.7 ± 918.3	916.4 ± 191.4	505.6 ± 148.5	151.0 ± 77.0
DEX:IPT	R	15318.0 ± 7166.6	3813.6 ± 1029.0	**1972.1 ± 296.9**	707.3 ± 276.9	223.2 ± 102.6
SAG:IPT	BS	2131.8 ± 1065.9	5060.4 ± 771.1	1885.3 ± 395.9	237.7 ± 49.9	141.8 ± 42.5
SAG:IPT	DC	1468.8 ± 453.3	4952.3 ± 1733.3	630.9 ± 42.7*	187.3 ± 24.2*	102.1 ± 27.0
SAG:IPT	D	**4268.2 ± 257.9**	**2089.2 ± 326.8***	**1440.2 ± 141.9**	**955.9 ± 168.0**	**312.5 ± 36.7**
SAG:IPT	RC	1568.7 ± 784.4	690.6 ± 20.3*	871.6 ± 89.6	363.9 ± 23.9	144.9 ± 43.5
SAG:IPT	R	**2925.6 ± 527.7***	**2749.8 ± 170.4**	**1604.4 ± 246.1**	375.5 ± 78.9	146.6 ± 12.3*
WT	BS	3719.6 ± 1859.8	4578.4 ± 667.4	1478.5 ± 198.6	261.9 ± 65.5	192.3 ± 57.7
Col-0 CK	DC	2326.4 ± 771.9	1626.8 ± 569.4*	1466.6 ± 64.3*	425.6 ± 17.2	194.0 ± 26.4
Col-0 CK	D	2850.0 ± 358.7	2021.4 ± 651.9	1459.7 ± 49.5	**934.3 ± 64.3***	**344.4 ± 48.7**
Col-0 CK	RC	1587.7 ± 320.6*	443.7 ± 73.2*	946.2 ± 160.4	469.7 ± 47.6*	168.1 ± 4.5
Col-0 CK	R	**4599.7 ± 811.8**	**2039.1 ± 142.1**	**1513.9 ± 94.3**	431.9 ± 25.8	156.5 ± 10.3*

Figure S1. Rosette area of WT, 35S:CKX, DEX:CKX, DEX:IPT, SAG:IPT plants, and Col-0 after *meta*-topolin treatment (Col-0 CK) in mm^2. Drought period lasted from 25 to 38 DAS; recovery period lasted from 38 to 44 DAS. Whiskers represent ± 1.5 x interquartile range (IQR).

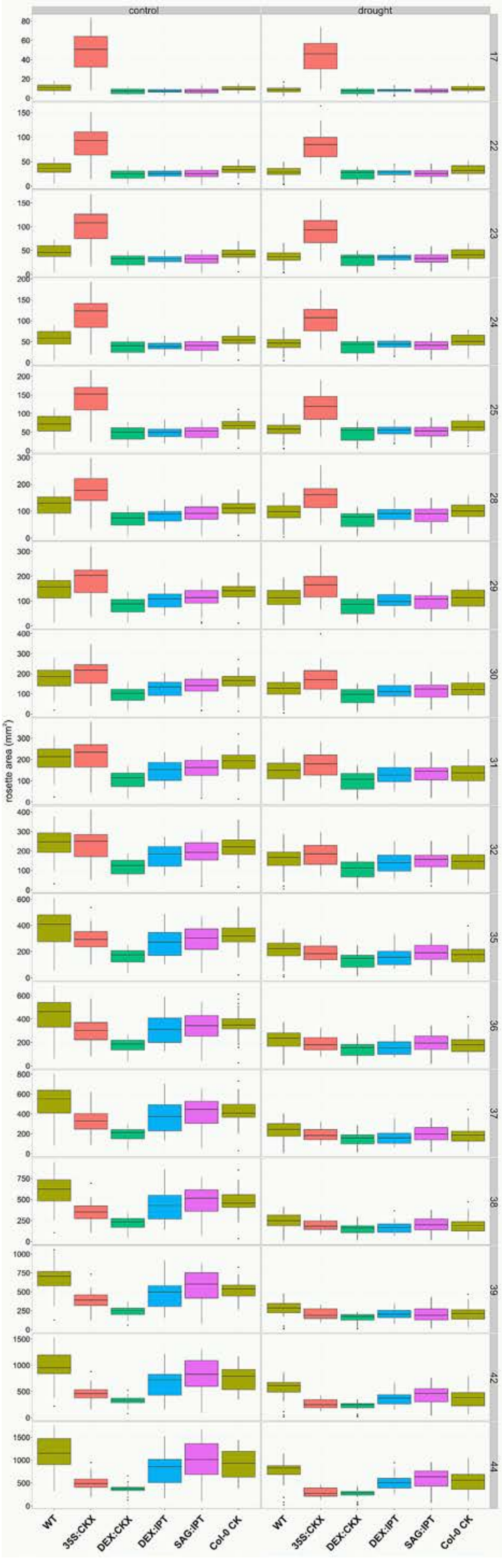
control
drought
rosette area (mm²)
17
22
23
24
25
28
29
30
31
32
35
36
37
38
39
42
44
WT
35S:CKX
DEX:CKX
DEX:IPT
SAG:IPT
Col-0 CK

Figure S2: Chlorophyll fluorescence parameter Fv/Fm changes in time. Before stress – 22 and 25 DAS; Drought – 28, 32, 35, 38 DAS; Recovery – 39, 42, 44 DAS. Data represent means ± SD (n = 6-13). Statistically significant differences ($p < 0.01$, two-sample t-test) between WT and a transformant of according variant (drought or control) in each time-point are marked by *; and between D/DC or R/RC variants within each genotype in each time-point are marked by +.

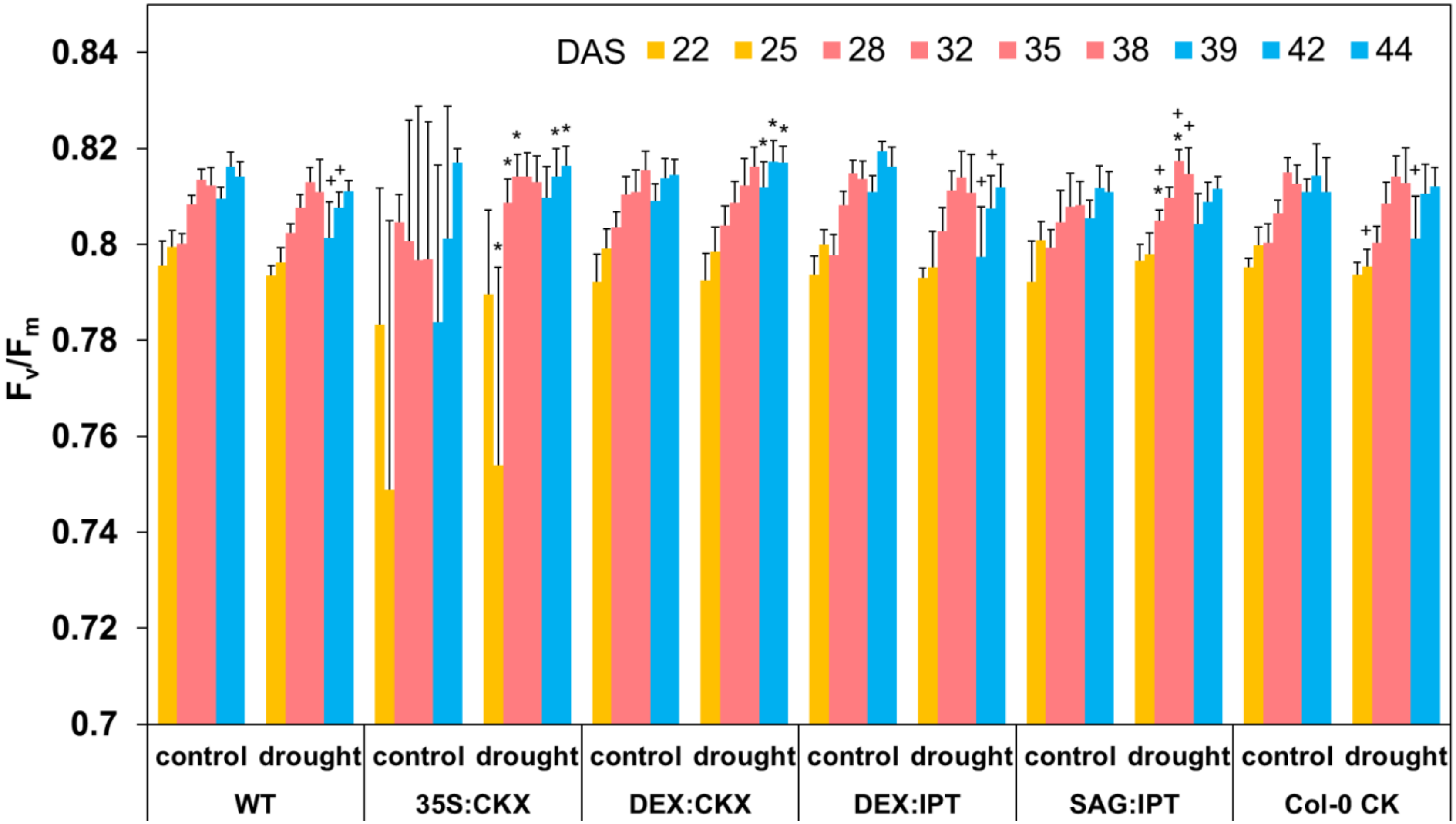

Supplement: Supplementary file 1 [file Data_Sheet_1.pdf]
